# Nanometre-scale probing of spin waves using single-electron spins

**DOI:** 10.1038/ncomms8886

**Published:** 2015-08-07

**Authors:** Toeno van der Sar, Francesco Casola, Ronald Walsworth, Amir Yacoby

**Affiliations:** 1 Department of Physics, Harvard University, 17 Oxford St., Cambridge, Massachusetts 02138, USA; 2 Harvard-Smithsonian Centre for Astrophysics, 60 Garden St., Cambridge, Massachusetts 02138, USA

## Abstract

Pushing the frontiers of condensed-matter magnetism requires the development of tools that provide real-space, few-nanometre-scale probing of correlated-electron magnetic excitations under ambient conditions. Here we present a practical approach to meet this challenge, using magnetometry based on single nitrogen-vacancy centres in diamond. We focus on spin-wave excitations in a ferromagnetic microdisc, and demonstrate local, quantitative and phase-sensitive detection of the spin-wave magnetic field at ∼50 nm from the disc. We map the magnetic-field dependence of spin-wave excitations by detecting the associated local reduction in the disc's longitudinal magnetization. In addition, we characterize the spin–noise spectrum by nitrogen-vacancy spin relaxometry, finding excellent agreement with a general analytical description of the stray fields produced by spin–spin correlations in a 2D magnetic system. These complementary measurement modalities pave the way towards imaging the local excitations of systems such as ferromagnets and antiferromagnets, skyrmions, atomically assembled quantum magnets, and spin ice.

## Abstract (web summary)

Exploring magnetic excitations and spin textures on the nanoscale may lead to new spintronic technologies and new understanding of condensed matter. Here, the authors demonstrate the potential of single-electron spins in diamond to image such excitations by characterizing spin waves in a ferromagnetic microdisc.

## Graphical abstract

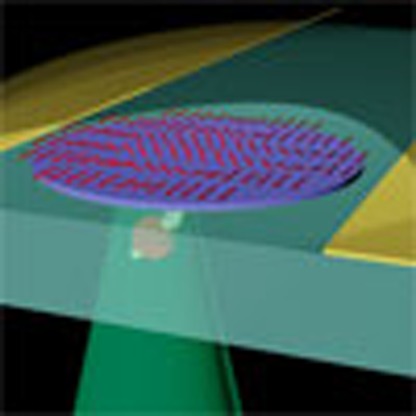

Correlated-electron systems support a wealth of magnetic excitations, ranging from conventional spin waves to exotic fractional excitations in low-dimensional or geometrically frustrated spin systems^1^,^2^. Probing such excitations on nanometre length scales is essential for unravelling the underlying physics and developing new spintronic nanodevices^3^,^4^,^5^,^6^. Despite recent progress with real-space techniques^7^,^8^,^9^,^10^,^11^,^12^,^13^, a wide range of interesting magnetic phenomena in correlated-electron materials remains experimentally inaccessible because of the required combination of resolution, magnetic-field sensitivity and environmental compatibility.

The *S*=1 electronic spin of the nitrogen-vacancy (NV) centre in diamond is an atom-sized magnetic field sensor that can be brought within a few nanometres of a sample and readily interrogated with optically detected magnetic resonance^14^. NV centre magnetometry^15^,^16^ has provided unprecedented room-temperature magnetic imaging with nanometre-scale resolution^14^,^17^,^18^,^19^ and single-proton-spin sensitivity^20^, and has been used to study nanoscale biomagnetism^21^,^22^. However, NV centres have only recently emerged as probes of collective spin dynamics in correlated-electron systems^19^,^23^. In this work, we demonstrate that single-NV magnetic imaging is a powerful tool for nanometre-scale, quantitative, and non-perturbative detection of spin-wave excitations. We present complementary measurement techniques to study spin-wave excitations over a broad range of magnetic fields and frequencies, as well as a method to characterize spin–spin correlations. These methods may be directly applied to open problems of current interest, such as real-space imaging of skyrmion core dynamics^24^ or imaging spin-wave excitations in atomically assembled magnets^10^ as a function of temperature.

## Results

### Spin waves in a ferromagnetic microdisc

**Figure 1:**
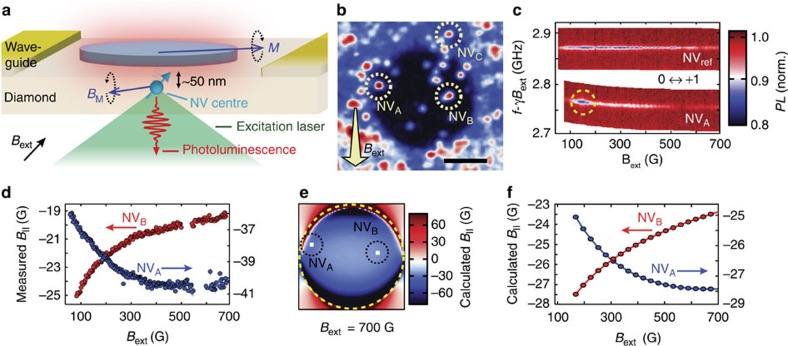
Nanoscale probing of the magnetic fields generated by a ferromagnetic microdisc. (**a**), As a model system to study magnetic excitations, we consider a ferromagnetic microdisc (Ni_81_Fe_19_, diameter 6 μm, thickness 30 nm) fabricated on top of a diamond surface. NV centres implanted at ∼50 nm below the surface sense the local magnetic fields *B*_M_ generated by the magnetization *M*. (**b**) Scanning confocal microscopy image showing a photoluminescence map of NV centres close to the disc. Scale bar, 3 μm. The external static magnetic field *B*_ext_ is applied along the axis of target NV centres. (**c**) Optically detected electron spin resonance (ESR) traces of the *m*_s_=0↔+1 transition of NV_A_ (close to the disc) and NV_ref_ (at ∼11 μm from the disc centre), where *m*_s_ denotes the spin-projection onto the NV-axis, *f* is the drive frequency, and *γ*=2.8 MHz/G. From the ESR frequency of NV_ref_ we extract the external magnetic field *B*_ext_. From the difference between NV_A_ and NV_ref_ we extract the disc stray field at the site of NV_A_. The dashed circle indicates power broadening caused by amplification of the drive field by a spin-wave excitation. (**d**) Projection onto the NV axis *B*_∥_ of the measured disc stray field as a function of external field, showing opposite behaviour at the sites of NV_A_ and NV_B_. The sign of the field is relative to *B*_ext_. Error bars represent±1 standard deviation determined statistically from typically ≳100 repetitions of the same measurement. (**e**) Calculated spatial profile of the projection of the disc stray field onto the NV axis in a plane 50 nm below the disc. *B*_ext_=700 G. (**f**) Numerically calculated projection of the disc stray field onto the NV axis as a function of the external field at the sites of NV_A_ and NV_B_, in qualitative good agreement with the measurements in **d.**

As a model system, we consider a ferromagnetic microdisc (Ni_81_Fe_19_) fabricated on top of a diamond chip that contains NV centres implanted at ∼50 nm below the surface (Fig. 1a,b). We use an on-chip coplanar waveguide to generate microwave magnetic fields to control the NV spin state and to drive spin-wave excitations in the disc. We optically address individual NV centres using a scanning confocal microscope (Fig. 1b) and read out the NV spin state through spin-dependent photoluminescence (Supplementary Note 1).

### Characterizing the static magnetization

Characterization of the static magnetization forms the basis for understanding the excitations of a magnetic system. Using individual NV centres close to the disc, we locally characterize the magnetization as a function of an externally applied static magnetic field *B*_ext_ (see Methods). We measure the electron spin resonance (ESR) frequency of an NV centre close to the disc (NV_A_ in Fig. 1b) and a reference NV centre (NV_ref_) far from the disc (Fig. 1c). By comparing these ESR frequencies and knowing the NV gyromagnetic ratio *γ*=2.8 MHz G^−1^, we determine the stray magnetic field of the disc at the location of NV_A_ (see Methods). Figure 1d shows the projection of this disc stray field onto the NV axis, *B*_∥_, as a function of *B*_ext_.

The local nature of the disc's magnetization becomes clear by comparing the measured disc stray field at two locations (NV_A_ and NV_B_, Fig. 1b). At both NV_A_ and NV_B_, this field opposes the external field (Fig. 1d), as expected from a numerically calculated spatial field profile based on a micromagnetics simulation of the disc's magnetization (Fig. 1e, see also Supplementary Note 2 and Supplementary Fig. 1). However, as *B*_ext_ is decreased, the change in the disc stray field at NV_A_ is remarkably opposite to that at NV_B_ (Fig. 1d). This behaviour is qualitatively in good agreement with numerical simulations of the disc's magnetization and the associated disc stray field as a function of *B*_ext_ (Fig. 1f). These calculations indicate that as *B*_ext_ is decreased, the magnetization becomes less homogeneous, with spins at the disc's edge reorienting first. The opposite behaviour of the disc stray field at NV_A_ and NV_B_ is a direct result of the differently varying local magnetization (Methods), and would not be observed in a far-field measurement.

### Resonant detection of spin-wave excitations

**Figure 2:**
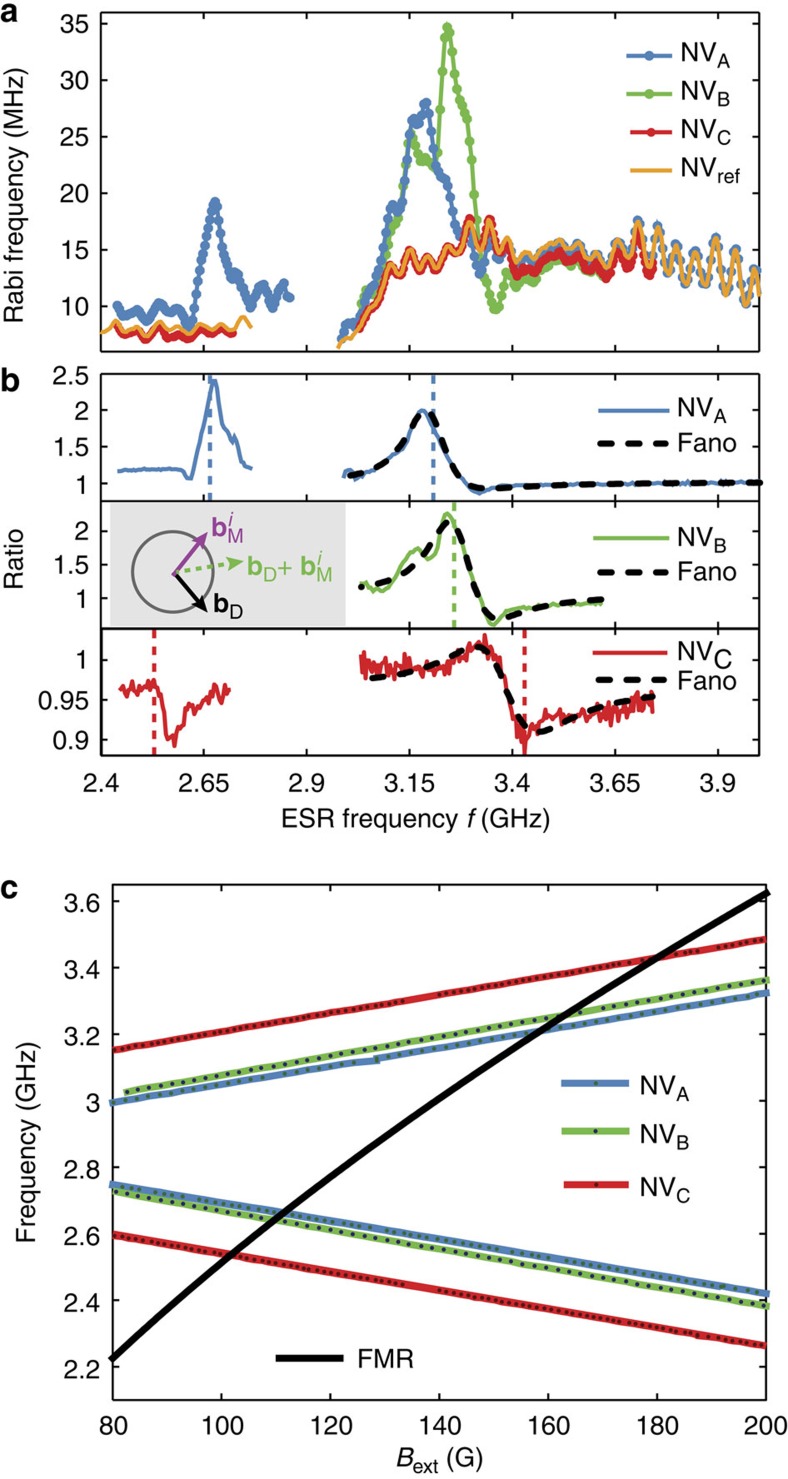
Resonant detection of driven spin-wave excitations. (**a**), Measured Rabi frequency as a function of ESR frequency for three NV centres close to the disc (NV_A_, NV_B_ and NV_C_, see Fig. 1b) and reference NV centre NV_ref_ far from the disc. We tune the NV ESR frequency using the external magnetic field *B*_ext_ and drive Rabi oscillations by applying a MW magnetic field at the ESR frequency. The ESR frequency range below (above) 2.87 GHz corresponds to the *m*_s_=0↔−1 (*m*_s_=0↔+1) transition. (**b**), Ratio of the Rabi frequency of NV_*i*_ (*i*=A,B,C) over the Rabi frequency of NV_ref_, as measured in **a**. Pronounced resonances are visible where the NV centre ESR frequency matches the numerically calculated ferromagnetic resonance (FMR) of the disc, which occurs at the vertical dashed lines (see **c**). The grey inset depicts the interference between the a.c. magnetic field generated by a spin-wave excitation 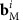 at the site of NV_i_ (*i*=A,B,C) and the driving field **b**_D_ in a frame rotating at the NV centre ESR frequency. (**c**) Measured ESR frequencies and numerically calculated FMR frequency as a function of external magnetic field *B*_ext_.

Spin-wave excitations consist of collectively precessing spins in a magnetically ordered system. It was recently proposed^25^ that detection of the time-varying stray magnetic fields generated by spin-wave excitations in small ferromagnets may be exploited to strongly amplify the sensitivity of single NV-centre magnetometry. Here we employ a resonant detection technique to locally sense the spin-wave stray magnetic field, demonstrating the first single-spin detection of on-chip magnetic-field amplification by a ferromagnet. We apply a microwave (MW) magnetic field to drive spin-wave excitations in the disc, choosing the MW frequency such that it is resonant with the ESR frequency of a target NV centre. The spins in the disc respond and generate a magnetic field 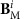 at the site of NV_*i*_ which interferes with the drive field **B**_D_. Transformed into a frame, rotating at the ESR frequency *f*, these fields are represented by 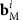 and **b**_D_ respectively, and sum (inset Fig. 2b) to give the total field 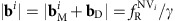 driving spin rotations (Rabi oscillations) of NV_*i*_ at a rate 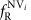. As we tune the NV ESR frequency using *B*_ext_, we observe a striking difference between the Rabi frequency of nearby NV centres (NV_*i*=A,B,C_, see Fig. 1b) and the Rabi frequency 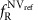 of a far-away, reference NV_ref_ (Fig. 2a). This difference becomes even clearer by plotting the ratio 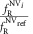, which corrects for any frequency-dependent delivery of MWs through our setup (Fig. 2b).

Numerical calculations of the spin-wave spectrum of the disc (Supplementary Note 2) indicate that the resonances in Fig. 2b occur when the NV centre ESR frequency matches the frequency of the lowest order spin-wave resonance of the disc (Fig. 2c). This mode—the ferromagnetic resonance (FMR)—is efficiently excited because the driving field is spatially uniform (Supplementary Note 2 and Supplementary Fig. 2). The observed resonance is described by


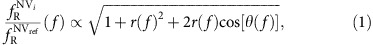


where 
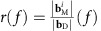
, and *θ*( *f* ) is the angle between 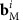 and **b**_D_ determined by the dynamic susceptibility of the ferromagnet and the location of the NV centre. To illustrate the validity of this model, we fit the resonances in Fig. 2b using equation (1), assuming a simple, single-mode damped oscillator response for *r*( *f* ) and *θ*( *f* ) (Supplementary Note 3). The resulting Fano-lineshape accurately describes the observed interference for NV_A_ and NV_C_, demonstrating that this technique is sensitive to both the amplitude and phase of the spin-wave magnetic field. Possible deviations from this model, such as the double-peak structure of NV_B_, may result from frequency dependence of the spatial profile of the spin-wave excitation or fabrication-related imperfections. The amplification of the MW field also explains the power broadening of the ESR spectra at low applied magnetic fields *B*_ext_, as observed in Fig. 1c.

### Non-resonant detection of spin-wave excitations

**Figure 3:**
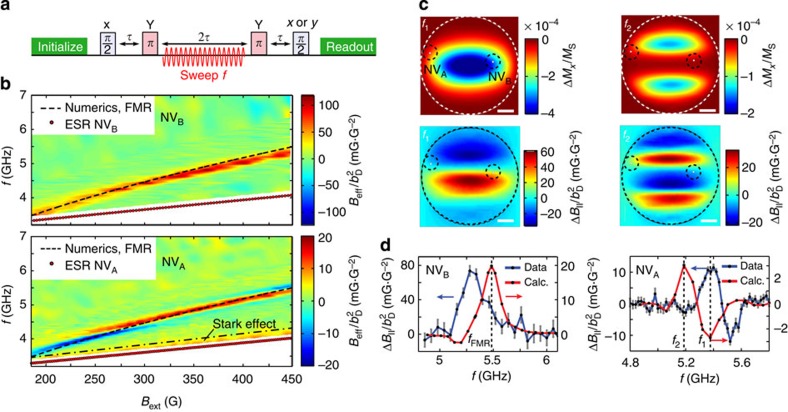
Non-resonant, field-dependent detection of driven spin-wave excitations. (**a**) Measurement sequence. The first *π*/2 pulse prepares an NV spin superposition, which is input into an echo sequence with two *π* pulses. Synchronized with this sequence, we apply microwave (MW) driving at frequency *f* during the central 2*τ* period of free evolution to excite spin-wave excitations in the disc. We read out the final phase *ϕ* of the NV spin state by measuring the projection on the *x* and *y* axis. (**b**) Experimentally determined effective magnetic field *B*_eff_ at NV_B_ and NV_A_ as a function of MW-driving frequency *f* and external static magnetic field *B*_ext_. *B*_eff_ is normalized by the square of the drive field 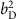 measured on-chip using NV_ref_. The dashed line is a numerical calculation of the ferromagnetic resonance (FMR) of the disc. For NV_A_, the a.c. Stark effect is visible as an enhanced signal in an ∼300-MHz frequency band between the ESR frequency and the dashed-dotted line over the entire magnetic field range. We use the sign of the Stark effect to determine the sign of *B*_eff_ in these measurements. Scale bar, 1 μm. (**c**) Numerically calculated spatial profile of the time-averaged change in the disc's longitudinal magnetization Δ*M*_*x*_ relative to the saturation magnetization *M*_S_ under spatially uniform driving with a 5 G MW field and the associated stray magnetic field Δ*B*_∥_ in the NV-plane, for two MW frequencies close to the FMR *f*_1_=*f*_FMR_−0.1 GHz and *f*_2_=*f*_FMR_−0.3 GHz. We use an external static field *B*_ext_=450 G, corresponding to the highest field used in the measurements in **b**, at which we expect the disc magnetization to be the most homogeneous and resemblant of the calculated magnetization. (**d**) Comparison of measured and calculated FMR lineshapes at *B*_ext_=450 G for NV_B_ and NV_A_. The sign, amplitude and width of the lineshapes accurately match the calculations. The 4% difference in frequency presumably results from a difference in the disc's saturation magnetization and/or fabrication-related imperfections. Error bars represent ±1 standard deviation determined statistically from typically≳100 repetitions of the same measurement.

Characterizing the magnetic excitation spectrum in a correlated-electron system, as well as addressing other problems of interest such as imaging magnetic vortex^8^,^9^ or skyrmion core dynamics^24^, requires a detection scheme that operates over a broad frequency range. To this end, we developed an off-resonant detection technique that can detect a sample's spin dynamics even when the NV centre ESR frequency is far detuned from the frequency of these dynamics. The idea is to drive spin-wave excitations in the sample with a microwave magnetic field and detect the resulting change in the stray magnetic field by applying a multipulse sensing sequence to the NV centre (Fig. 3a)^26^.

To understand the off-resonant detection scheme in Fig. 3a, it is crucial to realize that during the excitation of a spin-wave resonance, the time-averaged longitudinal magnetization of the disc is reduced (because the precessing spins are tilted away from their equilibrium state), causing a change in the time-averaged disc stray field (Supplementary Fig. 3). By applying the MW driving only during the central 2*τ* period (Fig. 3a), the disc stray field is modulated in sync with the multipulse sensing sequence applied to the NV centre, leading to a phase shift *ϕ* on the final NV spin state. At the end of the sequence, we read out this phase and relate it to an effective magnetic field *B*_eff_=*ϕ*/(γ*T*) oriented along the NV axis and averaged over the duration *T* of the MW driving. We note that the excitation of a spin-wave resonance also generates rapidly oscillating magnetic fields with typical frequencies in the GHz range (recall Fig. 2). However, such frequencies are above the detection capabilities of the scheme in Fig. 3a, because it would require applying the NV spin-control pulses at GHz repetition rates^26^. Although an exceptional situation occurs for frequencies close to the NV ESR frequency, where the NV spin may pick up a phase through the dynamical (that is, a.c.) Stark effect^27^, the Stark effect quickly diminishes for increasing detuning with the NV ESR frequency and we estimate it to play a minor role in our measurements (Supplementary Note 4 and Supplementary Fig. 4).

On the application of the measurement scheme in Fig. 3a to NVA and NVB, we observe a clear resonance that agrees well with numerical calculations of the FMR frequency of the disc (Fig. 3b, Supplementary Note 2). In Fig. 3b, *B*_eff_ is normalized by the square of the drive field |**b**_D_|^2^ measured on-chip using NV_ref_ to correct for a frequency dependence in the setup transmission (Methods and Supplementary Note 5). The resonance follows a Kittel-like law 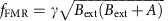, where *A* is a free parameter, characteristic of spin-wave excitations in a thin ferromagnet with in-plane magnetization. We therefore conclude that the observed resonance corresponds to the FMR of the disc. Furthermore, we observe striking differences in the lineshape of the resonances detected with NV_A_ and NV_B_ (Fig. 3b). To gain more insight into the origin of these differences, we now analyse the influence of the NV centre spatial location in these measurements.

Importantly, the reduction in time-averaged longitudinal magnetization associated with the excitation of a spin-wave mode is not homogeneous in space, but occurs within a specific spatial region of the disc in accordance with the spin-wave mode profile^7^. The location of an NV centre with respect to this profile determines the sign and magnitude of the corresponding change in magnetic field Δ*B*_∥_(*f*) that is felt by the NV centre (parallel to its axis). Because of the close proximity of the NV centres, Δ*B*_∥_(*f*) strongly depends on the NV-centre location (Fig. 3c). In addition, the spatial mode profile depends on frequency (Fig. 3c), affecting the lineshape of Δ*B*_∥_(*f*). At *B*_ext_=450 G we find a remarkably good agreement of the sign, width, and shape of the measured FMR signal with calculations (Fig. 3d) given geometrical uncertainties related to the optical resolution (∼400 nm), disc fabrication, NV implantation depth, and oxidation. However, we note that these calculations and/or our model do not account for the change in FMR lineshape observed at NV_A_ as we decrease *B*_ext_. Such strong sensitivity on location highlights the unique possibilities NV centres offer to study spin dynamics quantitatively and with nanometre-scale resolution.

### Detection of spin noise

**Figure 4:**
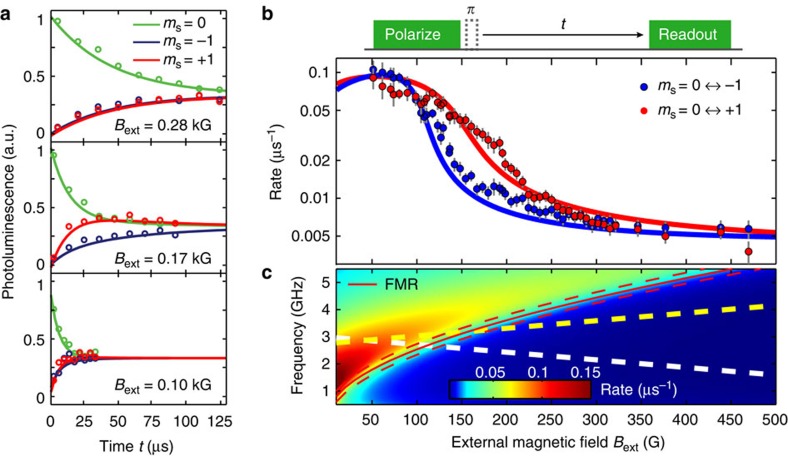
Probing the spin–noise spectrum. (**a**) Spin relaxation measurements for NV_A_ at three different external static magnetic fields *B*_ext_. The NV centre is prepared in each of its three spin eigenstates (*m*_s_=0, −1, +1), and the spin-dependent photoluminescence is measured as a function of waiting time *t*. We extract the *m*_s_=0↔+1 and the *m*_s_=0↔−1 relaxation rates by fitting with a three-level model. (**b**) Measured NV spin relaxation rates as a function of *B*_ext_. As we lower *B*_ext_, the *m*_s_=0↔+1 and the *m*_s_=0↔−1 relaxation rates consecutively increase by about an order of magnitude. Dots are measured data. Solid lines are fits based on a model of the magnetic noise spectrum at the site of the NV centre, from which we obtain *d*=35(5) nm. Error bars represent±1 standard deviation determined by the fit-uncertainty in the fitted relaxation rates. (**c**) Calculated magnetic noise spectrum at *d*=35 nm above an infinite magnetic plane as a function of external magnetic field *B*_ext_. The increasing NV spin-state relaxation rates for decreasing *B*_ext_ observed in **b** are due to the increase in noise spectral density at the NV ESR frequencies, which are denoted by the yellow and white dashed line for the *m*_s_=0↔+1 and *m*_s_=0↔−1 transition, respectively.

Spin noise contains valuable information about a system's magnetic excitation spectrum and is present even in the absence of driving^28^,^29^. Here we spectrally probe spin noise in the disc by measuring the spin relaxation rates of a proximal NV centre (NV_A_), which depend on the strength of the magnetic field generated by the spin noise at the NV centre ESR frequencies^30^,^31^. As we lower *B*_ext_ and thereby change the NV ESR frequencies relative to the spin–noise spectrum, we first find the *m*_s_=0↔+1 and then the *m*_s_=0↔−1 relaxation rate (where *m*_s_ denotes the projection of the spin state onto the NV axis) to increase by over an order of magnitude (Fig. 4a,b), indicating a marked increase in the noise at the ESR frequencies.

Qualitatively, this behaviour can be understood by realizing that at high magnetic field, the NV ESR frequencies are below the FMR frequency (recall Fig. 2c) and therefore in the gap of the spin-wave spectrum. In contrast, at low magnetic field the ESR frequencies are above the FMR frequency where spin waves do exist and generate noise. For a more quantitative understanding, we calculate the magnetic-noise spectrum at a distance *d* from an infinite, two-dimensional (2D) magnetic plane (Fig. 4c, see Methods). We use a general framework describing the noise spectrum at the site of a sensor spin in terms of the spin–spin susceptibility and a k-space filter function associated with the dipolar coupling to the spins in the magnet (see Methods). This filter function contains a kernel ∼*k*^2^*e*^−2*kd*^ that peaks at *k=*1*/d*, where *d* is the NV–disc distance and *k* is a wavenumber characterizing spatial fluctuations of the magnetization. This kernel reflects that a homogeneous magnetization (*k*=0) does not generate a magnetic field anywhere outside the plane. Likewise, the magnetic field generated by a spatially rapidly varying magnetization characterized by *k*>>*d* is exponentially suppressed. Clearly, the noise at the site of the NV centre is dominated by spin–spin correlations on the scale of *d*. Since the NV centre is far away from the disc's edges compared with *d*, we can approximate the disc by an infinite plane. Furthermore, we approximate the dynamic susceptibility as being dominated by exchange interactions because of the small value of *d* (Supplementary Note 6). This model excellently describes the measured increase in spin noise at the NV ESR frequencies as we lower *B*_ext_ (Fig. 4b). From fitting, we obtain *d*=35(5) nm (Supplementary Note 6). However, we note that the model underestimates the disc's thickness by more than an order of magnitude as detailed in Supplementary Note 6, possibly resulting from the model's 2D nature. It would be interesting to perform further experiments in which the NV-magnet distance and/or magnet thickness are varied to further develop and test the concepts of NV-relaxometry of spin waves. These relaxation measurements can be extended with 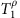 and *T*_2_ spectroscopy techniques^32^ to characterize a spin–noise spectrum over a range of frequencies at a fixed value of magnetic field.

## Discussion

In this work, the ferromagnetic microdisc was fabricated directly on top of the surface of a diamond containing NV centres. As such, the NV centres were fixed in space with respect to the disc. This configuration enables a determination of the lateral NV position to about 400 nm (set by the optical diffraction limit), and allows local detection of magnetic excitations with spatial variations on the scale of the ∼50-nm NV–disc distance. A variety of techniques may be used to improve the lateral imaging resolution to the few-nanometre scale: for example, optical superresolution methods^33^, real-space magnetic field gradients created by scanned magnetic tips^34^, and Fourier-imaging techniques similar to conventional MRI^35^. Because of the point-like nature of the NV centre, the ultimate imaging resolution is given by how close one can bring an NV centre to a sample and how well one can control its position with respect to the sample. As shown in several recent studies (see, for example, refs 20, 32), NV centres can readily exist at just a few nanometres below the diamond surface. Although the proximity of a metallic sample may quench fluorescence for emitter-metal distances below ∼10 nm (ref. 36), this should allow studies of, for example, a skyrmion lattice. Looking ahead, the complementary NV magnetometry techniques, demonstrated here for spin waves in a ferromagnetic disc, open up exciting possibilities to explore a wide variety of magnetic excitations in nanoscopic systems under ambient conditions. The techniques are directly applicable to imaging highly localized spin-wave excitations such as edge modes in nanomagnets^37^ or, when combined with THz sources^38^, high-energy excitations in patterned high-coercivity ferromagnets or in antiferromagnets. We envision nanometre-scale studies of magnetic vortices and skyrmions, atomically engineered quantum magnets^10^ and spin ice. In addition, these techniques can be applied to characterize the magnetic fields generated by edge currents in quantum Hall systems and topological insulators^13^.

## Methods

### Application of *B*_ext_

We apply the static external field *B*_ext_ along the axis of target NV centres to assure good optical spin contrast (Supplementary Note 7). *B*_ext_ is thus oriented at an angle of 54° with respect to the plane of the disc. Throughout this work, we select NV centres with equally oriented crystal axes. To avoid hysteresis in the disc, in all measurements we first apply a large field (*B*_ext_>700 G) and then sweep the field down in small steps.

### D.c. magnetometry

The ESR frequencies of an NV centre in a magnetic field **B** are determined by the Hamiltonian 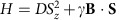, where *S*_*i*=*x*,*y*,*z*_ are Pauli spin matrices for a spin 1, *D* is the zero-field splitting, and *z* denotes the direction of the NV centre crystal axis. We use this Hamiltonian to calculate the projection of the magnetic field onto the NV-axis from the measured ESR frequencies (Fig. 1c,d), as described in detail in Supplementary Note 7.

### Normalization procedure

To obtain the signal in Fig. 3, we apply the pulse sequence in Fig. 3a and normalize the photoluminescence (PL) on spin readout using two reference measurements. In these measurements, we apply the sequence of Fig. 3a without the MW drive field and with the final *π*/2-pulse around the *x* or the –*x* axis, which yields minimum and maximum PL values. Using these bounds we normalize the PL measured at the end of the pulse sequence in Fig. 3a to obtain *B*_eff_ (Supplementary Fig. 5). We then divide *B*_eff_ by the square of the driving field |**b**_D_|^2^, which we independently determine by measuring the Rabi frequency of NV_ref_ as a function of the ESR frequency (Supplementary Fig. 6). The measured linear scaling of *B*_eff_ with the MW-source power validates this normalization procedure (Supplementary Fig. 7). The normalization is described in detail in Supplementary Note 5.

### Stray-field characterization of magnetization and spin noise

In this section, we describe the properties of stray-field magnetometry of magnetization and spin noise which are relevant for the experiments presented in this work. In particular, we will show that the NV centre probes the spatial variations in the magnetization on the scale of the NV–disc distance, and we will derive the model used for the calculations of the field-dependent magnetic noise spectrum at the NV-site shown in Fig. 4 (for more details, see Supplementary Note 6).

An NV spin at a distance *d* from the surface of the disc is mostly sensitive to local variations in the magnetization on the scale of *d*. Intuitively, this can be easily understood: on one hand, a homogeneously magnetized infinite plane generates no stray field. On the other hand, the stray field generated by variations in the magnetization on a scale much smaller than *d* averages out at a distance *d*. More formally, it can be shown that the stray field **B**(**r**_0_) at position **r**_0_=(**ρ**_0_,*d*) (Supplementary Fig. 7) produced by a certain 2D spin texture **S**(**ρ**_j_) is given by:


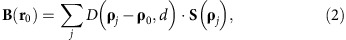


with *D*(**ρ**_j_ -**ρ**_0_,*d*) being the dipolar tensor. We note that by ‘two-dimensional spin texture' we imply a spin texture that varies in the plane but not along the thickness of the film. We consider a thin magnetic film having a saturation magnetization *M*_s_ and thickness *t*. We move to the continuous limit by calling Γ=*M*_S_*t*/(*g*_L_*μ*_B_*S*) the number of magnetic dipoles per unit surface. Here *μ*_B_ is the Bohr magneton, *g*_L_ the Landé *g*-factor of the local spin *S*. We obtain:


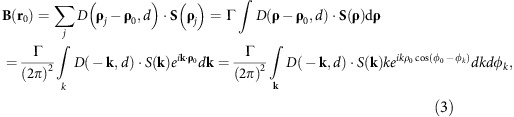


where **k** is a 2D vector in reciprocal space, and *φ*_0_–*φ*_*k*_ is the angle between the in-plane **ρ**_0_ and the **k** vector. Provided with such a formalism, we adopt cylindrical coordinates and compute the Fourier transform of the components of the dipolar tensor:


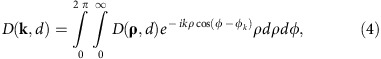


where **S**(**k**) is the spatial Fourier transform of **S**(**ρ**), *φ–φ*_*k*_ is the angle between **ρ** and the **k** vector, and *φ*_*k*_ is the angle between **k** and *z* (Supplementary Fig. 8). We see that stray-field detection works as a spatial Fourier filter, with a kernel given by:


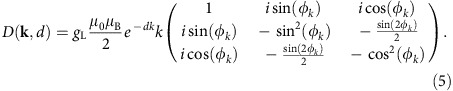


The NV-centre stray-field sensor is point-like, contrary to, for example, nano-SQUIDs or Magnetic-Resonance Force Microscopy probes. Therefore, the stray field computed with equation (3), after a proper projection along the NV axis, directly describes how spatial modulations of the local magnetization couple to the NV spin. We obtain the following general set of conclusions, in principle valid for any **S**(**ρ**) distribution. First, all the elements in the kernel contain the term *k* exp(-*dk*), which peaks at *k*=*1/d*. Stray-field magnetometry is insensitive to Fourier components of the magnetization whose spatial frequency coincides with the condition *D*(**k**,*d*)=0. Accordingly, an NV center cannot detect stray field originating from a uniformly magnetized (*k=0*) surface or from a spin structure that varies in space within distances much shorter than *d*. The region of maximum sensitivity corresponds to wavevectors *k*∼1*/d*, which in a 2D region of k-space defines an annulus. We refer to this region in k-space as detection annulus of the technique. Second, the in-plane stray-field component orthogonal to the wavevector **k** is always zero. We note that in equation (3) we have assumed that all the magnetic moments along the thickness *t* have the same distance *d* from the NV centre.

The formalism just described can be used to derive a general expression for the stray magnetic-field noise generated by spin noise in a thin magnetic film. Spin fluctuations δ**S**_j_(*t*) in the disc will produce a time-dependent field δ**B′**(**r**_0_,*t*) at the NV site, which can be written as:


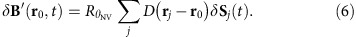


Here we inserted a rotation matrix 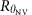 to express the field in an *x*′*y*′*z*′ frame which has the *z*′ axis parallel to the NV axis (that is, the *x*′*y*′*z*′ frame is in general rotated by an angle *θ* around the *y* axis with respect to the *xyz* frame, see Supplementary Fig. 8). We first compute the spectral density of the stray magnetic-field noise at energy *ω*_*α,β*_ along a general direction η:


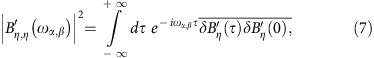


which has units of T^2^Hz^−1^. Here 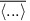 denotes an ensemble average over the magnet's spin degree of freedom. An expression for the stray magnetic-field noise can be obtained by inserting equation (6) into equation (7):


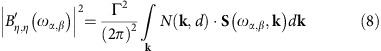


where we have defined **S**={*S*^*x,x*^*,S*^*y,y*^*,S*^*z,z*^}, with:


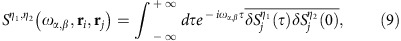


and


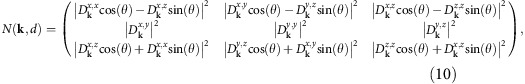


and the 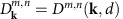 are the matrix elements of equation (5).

The matrix *N*(**k**,*d*) filters in ***k***-space the spin fluctuations of the magnetic thin film. Note that the integral in equation (8) contains a kernel *k*^*2*^exp(*−2dk*) for all the components of the *N*(**k**,*d*) matrix. The detection annulus changes therefore slightly with respect to the case of static magnetometry.

The model linking the relaxation rates of the NV centre to the spin–noise in the disc is discussed in Supplementary Note 6. In particular, the field-dependent noise spectrum and associated NV-relaxation rates 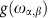 shown in Fig. 4c are obtained from the expression:


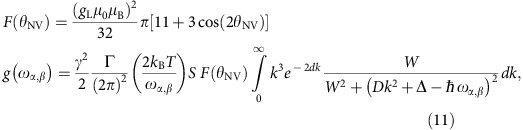


where *T*=300 K is the temperature, 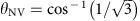, *W* is the width of the FMR excitation, Δ its field-dependent gap, *D* the spin stiffness and *S* the value of the local spin.

## Additional information

**How to cite this article:** van der Sar, T. *et al*. Nanometre-scale probing of spin waves using single-electron spins. *Nat. Commun.* 6:7886 doi: 10.1038/ncomms8886 (2015).

## Supplementary Material

Supplementary Information Supplementary Figures 1-11, Supplementary Notes 1-7 and Supplementary References
