# Erratum: Nanometre-scale probing of spin waves using single electron spins

**DOI:** 10.1038/ncomms9426

**Published:** 2015-10-07

**Authors:** Toeno van der Sar, Francesco Casola, Ronald Walsworth, Amir Yacoby

Nature Communications 6: Article number: 788610.1038/ncomms8886 (2015); Published: 08072015; Updated: 10072015

The original version of this Article contained an error in the phrase ‘single electron spins' in the title. This has now been corrected in both the PDF and HTML versions of the Article.

