# Supplementary material for: Nanometre-scale probing of spin waves using single-electron spins
Source: Nat Commun. 2015 Aug 7;6:7886. doi: 10.1038/ncomms8886 (PMC4918315; doi:10.1038/ncomms8886)
Supplement: Supplementary Information — Supplementary Figures 1-11, Supplementary Notes 1-7 and Supplementary References [file ncomms8886-s1.pdf]

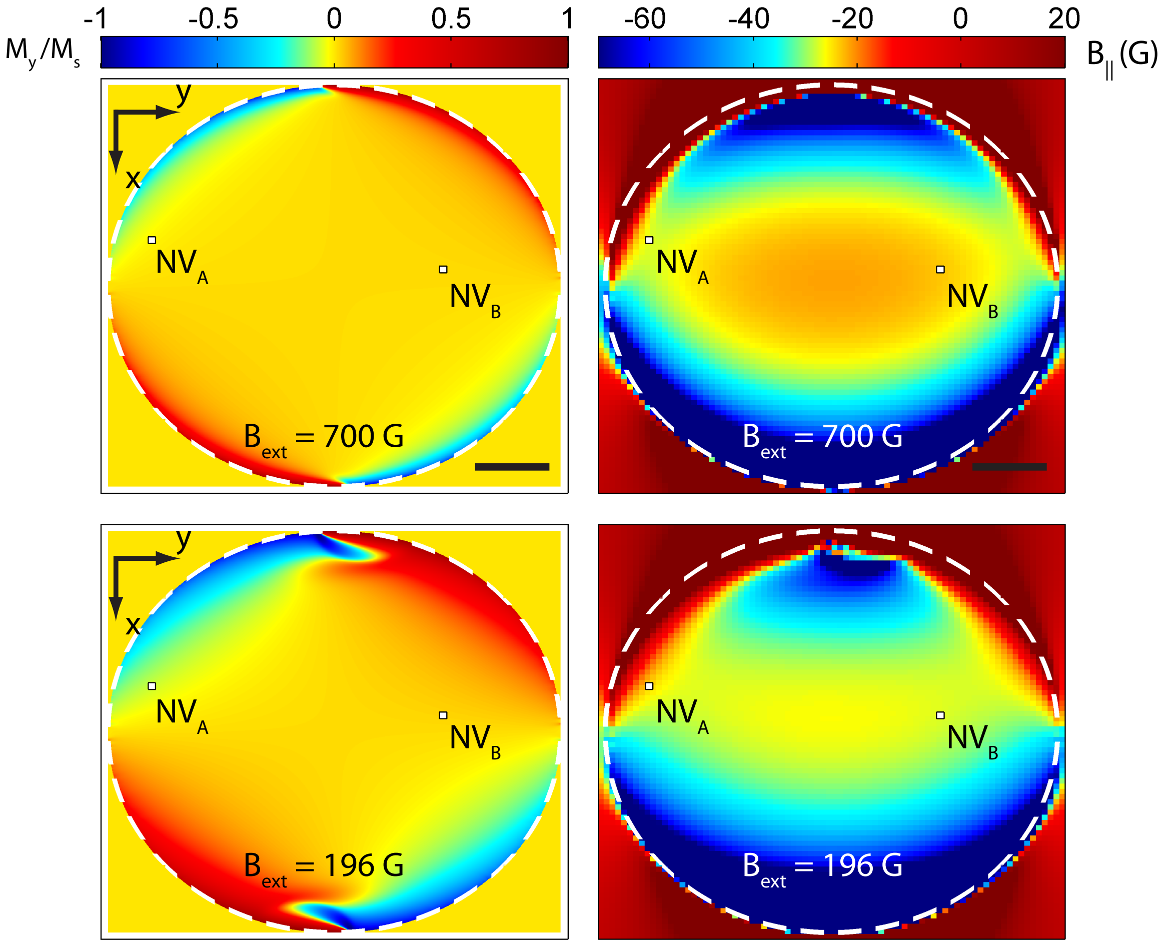

Supplementary Figure 1. **Micromagnetics simulation of spatial magnetization profiles at two different fields (left) and the associated calculated stray-field in the NV-plane (right).** Left: magnitude of the in-plane component of the magnetization perpendicular to the equilibrium direction (x) at $B = 700$ F (top), and $B = 196$ G (bottom). Right: associated magnetic field at a distance of 50 nm from the disc (the NV plane)

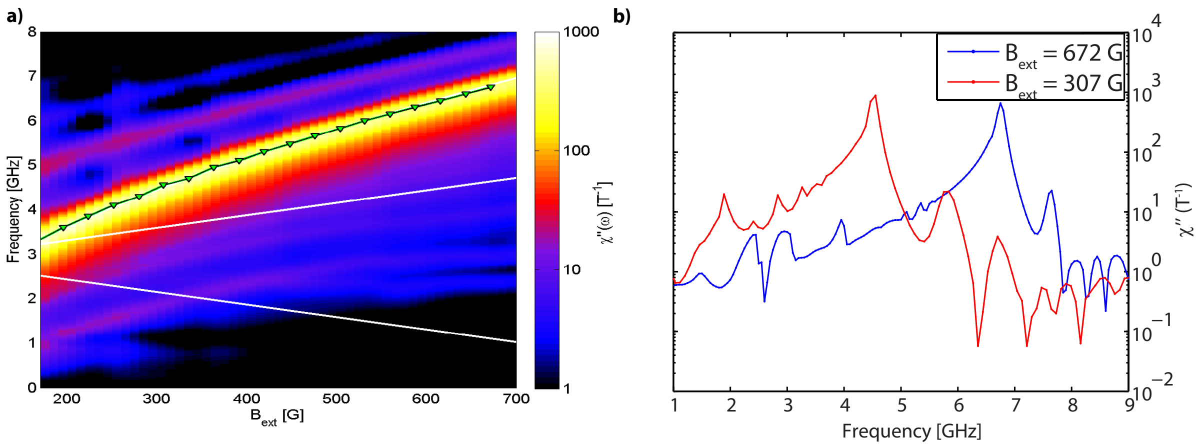

Supplementary Figure 2. **Linear transverse spin response of a Permalloy disc of 6μm in diameter a,** Colormap combining several $\chi''_{\perp}(\omega, B_{\rm ext})$, where the value of $B_{\rm ext}$ is the magnitude of the external field applied along the NV-axis direction. NV transitions are shown with white solid lines. Green triangles show the position of the uniform mode. **b,** Detail of $\chi''_{\perp}(\omega, B_{\rm ext})$ at the fields $B_{\rm ext} = 672$ and 307 G.

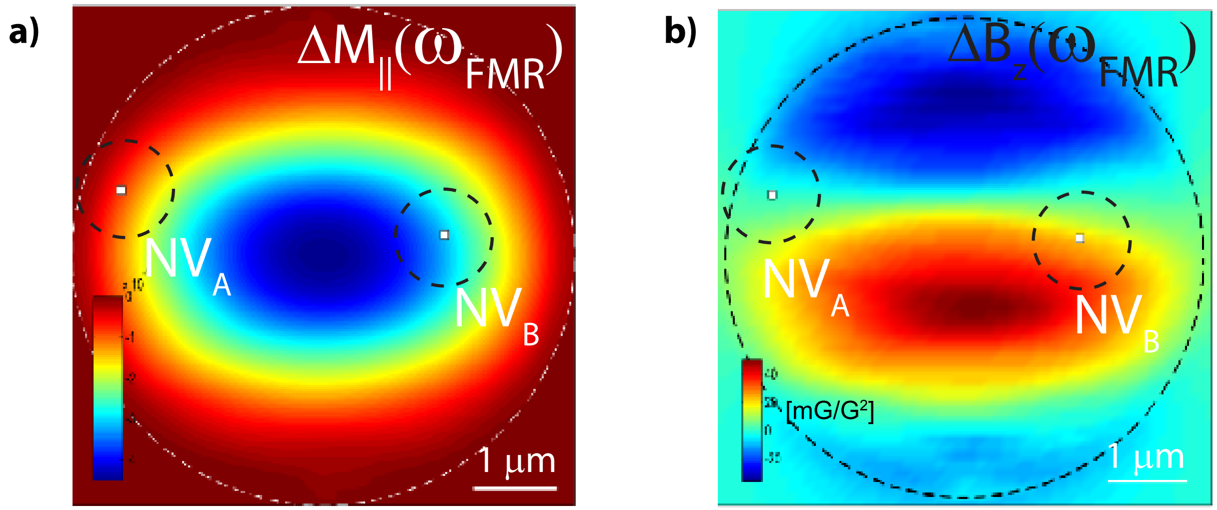

Supplementary Figure 3. **Time-averaged variation of the longitudinal magnetization and resulting stray field at the frequency $\omega_{\rm FMR}$ of the uniform mode. a,** In-plane component $\Delta M_{\|}$ of the time-averaged quantity $\langle \delta m_{\|,i}(t) \rangle \mathbf{u}_{\|,i}$. Magnetization is normalized to M$_s$. **b,** Stray magnetic field along the NV-axis direction originating from the local magnetization $\langle \delta m_{\|,i}(t) \rangle \mathbf{u}_{\|,i}$. The stray field has been normalized by the drive field. The NV depth has been taken to be 50 nm.

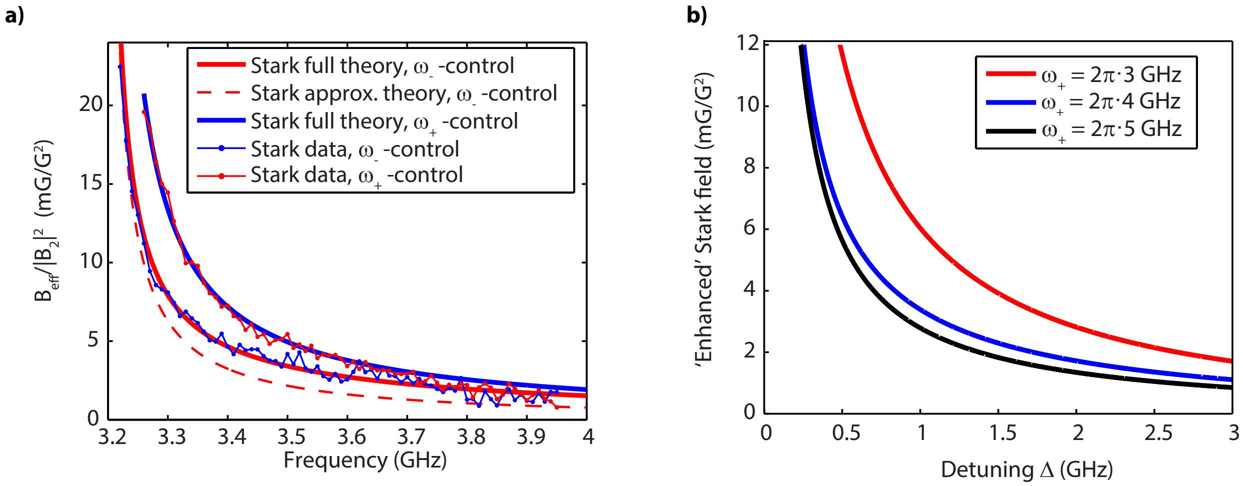

Supplementary Figure 4. Analysis of the Stark shift, theory and experiment. a, Effective Stark field B_{eff} as a function of the frequency of the off-resonant driving field at $B_{ext} =$ 110 G, where $\omega_+ = 2\pi \cdot 3.19$ GHz. We used the measurement scheme depicted in Fig. 3a of the main text. The red (blue) dots correspond to NV-control pulses applied at the $0 \leftrightarrow -1$ ($0 \leftrightarrow +1$) transition. Solid lines represent the parameter-free theoretical prediction given by Supplementary Eq. (20). The dashed line is the result expected from Supplementary Eq. (21). Because at this low field we have $(\omega_- - \omega) \sim (\omega_+ - \omega)$, Supplementary Eq. (21) (dashed red line) does not correctly approximate the experimental results. We normalized the effective field by the square of the drive field B_2^2 (see main text). **b,** Effective Stark field, *multiplied by a factor 4*, as a function of the detuning $\Delta = \omega - \omega_+$ for NV-control pulses applied at the $0 \leftrightarrow -1$ transition. The factor four corresponds to the maximum observed enhanced Rabi amplitude in Fig.2 of the main text, i.e., max $\left[\frac{f_R^{NV_i}}{f_R^{NV_{ref}}}(f)\right]$. To estimate the contribution of the Stark effect at the FMR frequency, we assume that the FMR enhances the drive field by approximately the same factor for the measurements in Fig. 3b-c of the main text. As is visible in the figure, the expected Stark effect varies with splitting of the $|\pm 1\rangle$ NV state, i.e. with the value of the static applied field.

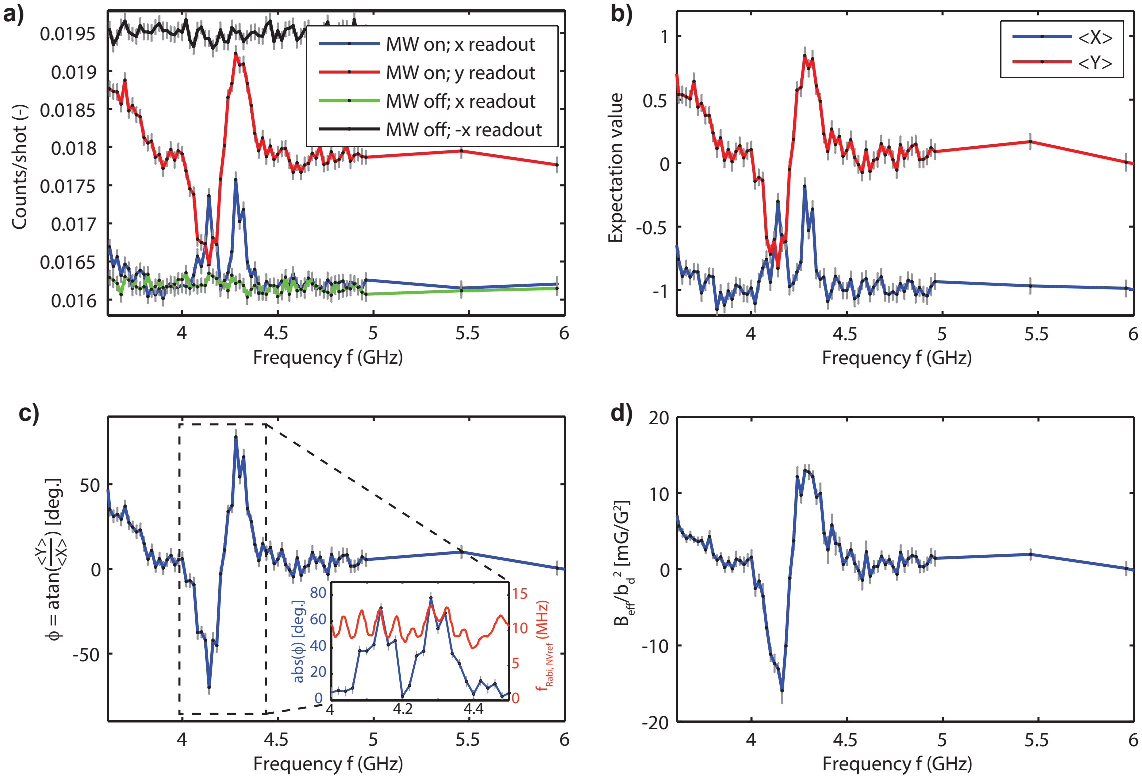

Supplementary Figure 5. **Example of the normalization procedure used to obtain main-text Fig. 3b and 3d: a frequency sweep at** $B_{ext} = 211$ **G on NV**$_A$**. a,** Raw data (details on pulse sequences in text) **b,** Spin expectation values, **c,** Phase of the final superposition. Inset: Absolute value of the phase and Rabi frequency of NV$_{ref}$ plotted in one graph. The dips/peaks in Rabi frequency clearly correspond to dips/peaks in ϕ. **d,** Effective field normalized by the power of the driving field.

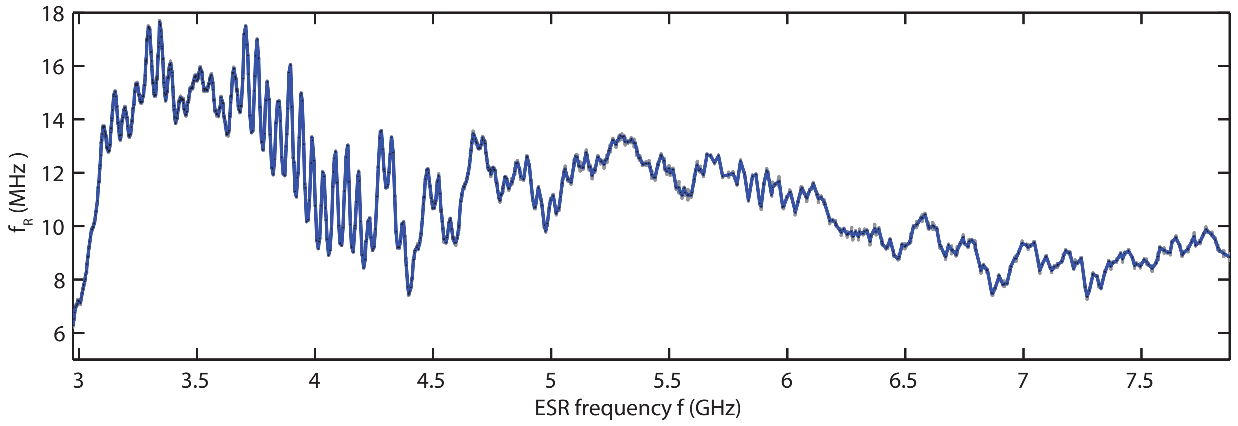

Supplementary Figure 6. **Characterization of the frequency-dependent transmission of microwaves through our setup.** Even though this measurement is performed with constant MW-source power, we observe a strongly frequency-dependent spin rotation rate (Rabi frequency f_R). Measuring the Rabi frequency is an excellent method to characterize in-situ how much MW power actually reaches the NV centre. From the Rabi frequency f_R, we obtain $|b_d| = f_R/\gamma$. Measurement performed on NV_{ref}, the ESR frequency is tuned using B_{ext}.

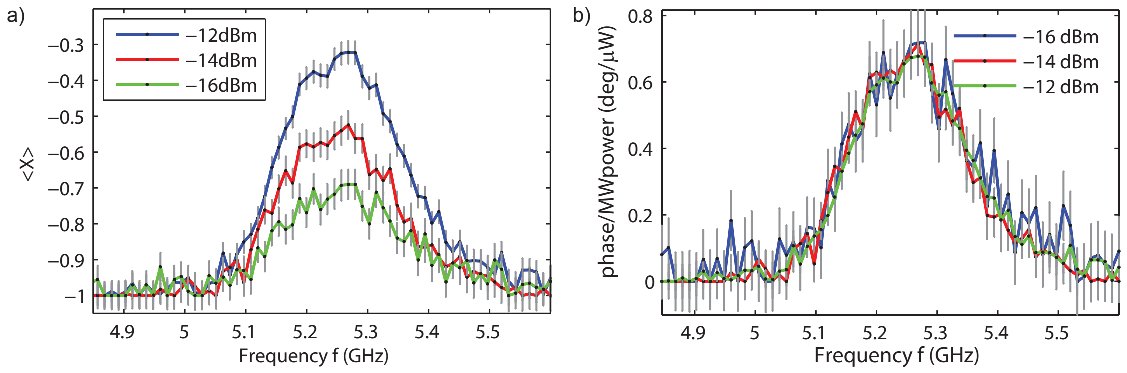

Supplementary Figure 7. **Linear scaling of phase** ϕ **with MW-source power. a,** FMR measured on NV_B for different MW powers at $B_{ext} = 450$ G. **c,** The phase divided by the MW-source power, illustrating the linear scaling with MW-source power.

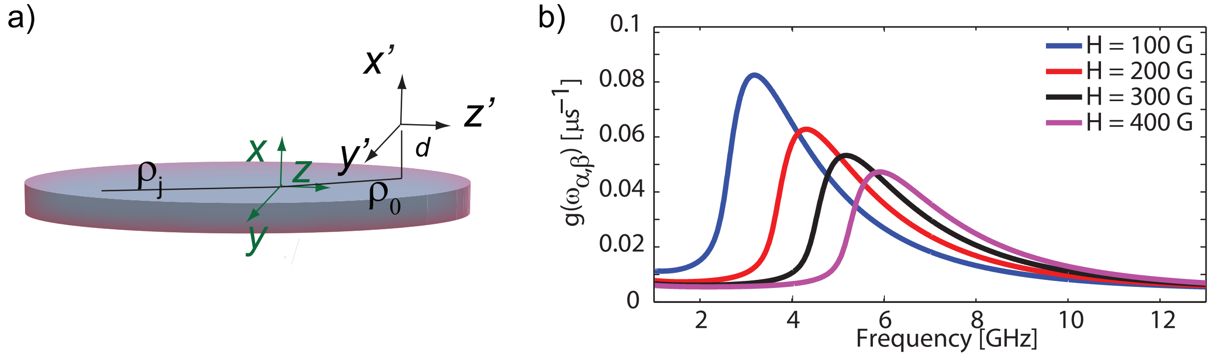

Supplementary Figure 8. **a,** Reference frame xyz for the disc and $x'y'z'$ for the stray-field probe. **b,** Field-dependence of the $g(\omega_{\alpha,\beta})$ rate for various magnetic fields, following Supplementary Eq. (33) and using the parameters extracted from the fit in Fig.4b of the main text. A field-independent constant rate was added to model the contribution of longitudinal spin fluctuations.

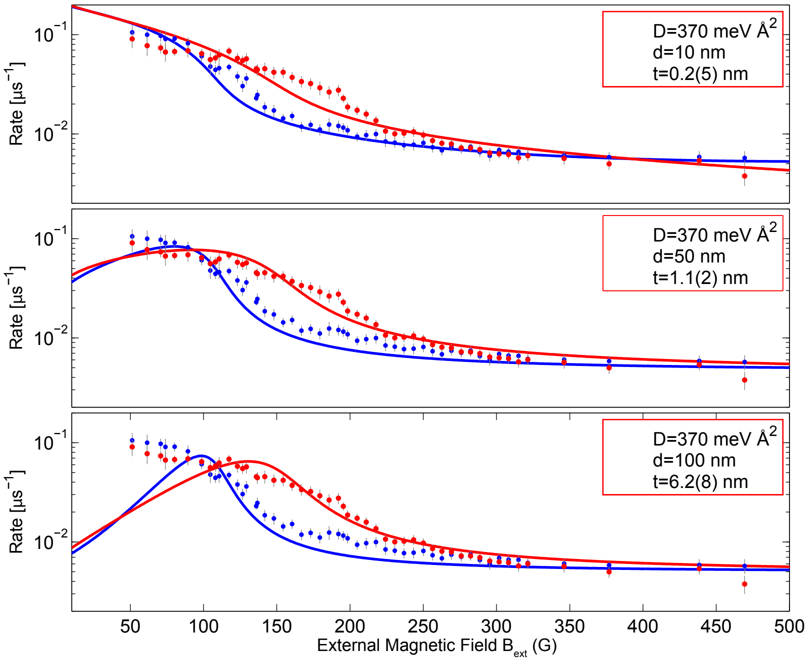

Supplementary Figure 9. **Dependence of the spin-noise model on the NV-disc distance** $\boldsymbol{d}$**.** The solid lines are fits of Supplementary Eq. (33) to the measured NVA spin relaxation rates also presented in Fig. 4b of the main text. During the fits, the parameter t and a field-independent offset $g_{||}$ accounting for the longitudinal spin fluctuations were released. The FMR line width W was taken from fits of the Fano interferences representing the resonant detection of the spin-wave excitations.

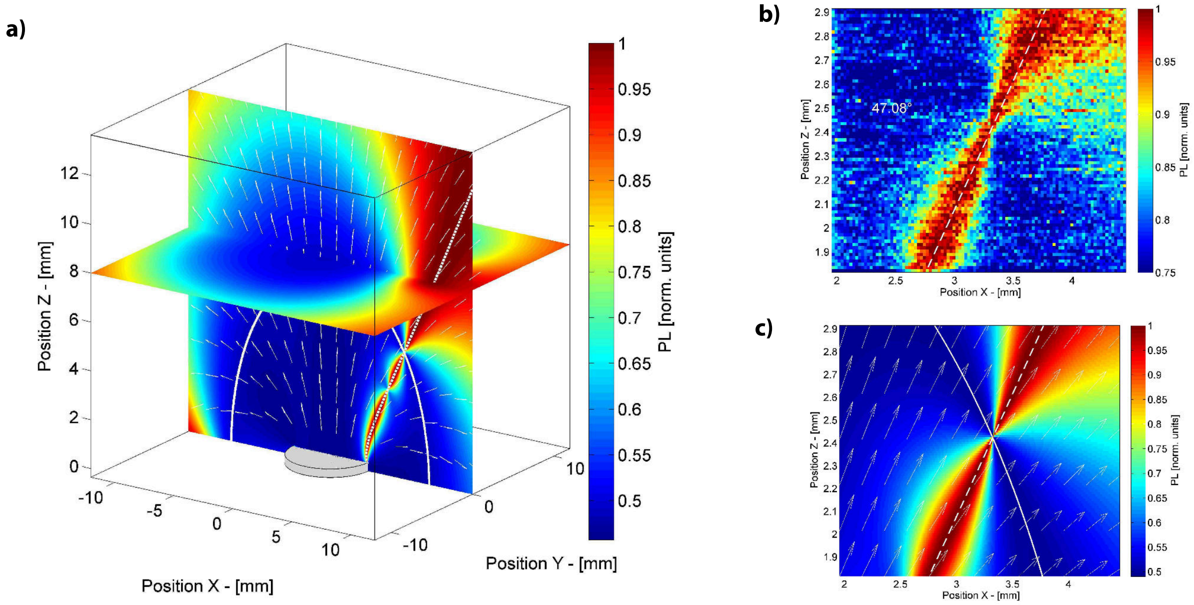

Supplementary Figure 10. **NV-centre photoluminescence (PL) as a function of magnet position.** Left: Numerically calculated PL for an NV centre in the field of a cylindrical NdFeB permanent magnet of diameter 6.35 mm and height 12.7 mm (the model magnet D48-N52 produced by K&J magnetics). The NV axis is oriented along the $[\sin(\theta_{\rm NV}), 0, \cos(\theta_{\rm NV})]$ direction, with $\theta_{\rm NV} = \arccos(1/\sqrt{3})$, and moved in space in the vicinity of the magnet's surface (gray disc). The origin of the reference frame represents the centre of the magnet's top surface. The white solid line marks the positions where the field is 514 G. The dashed white line marks the positions where the field is aligned with the NV axis. White arrows represent the directions of the field in the various positions. Right: Experimentally measured (top) and numerically computed (bottom) photoluminescence (PL) in the vicinity of the $\sim$ 500 G region. There, the PL is easily quenched because of the spin mixing corresponding to the level anti-crossing of the NV excited states.[1] The calculation/measurement refers to a NdFeB permanent magnet of diameter 3.175 mm and height 9.525 mm (model magnet D26-N52 produced by K&J magnetics).

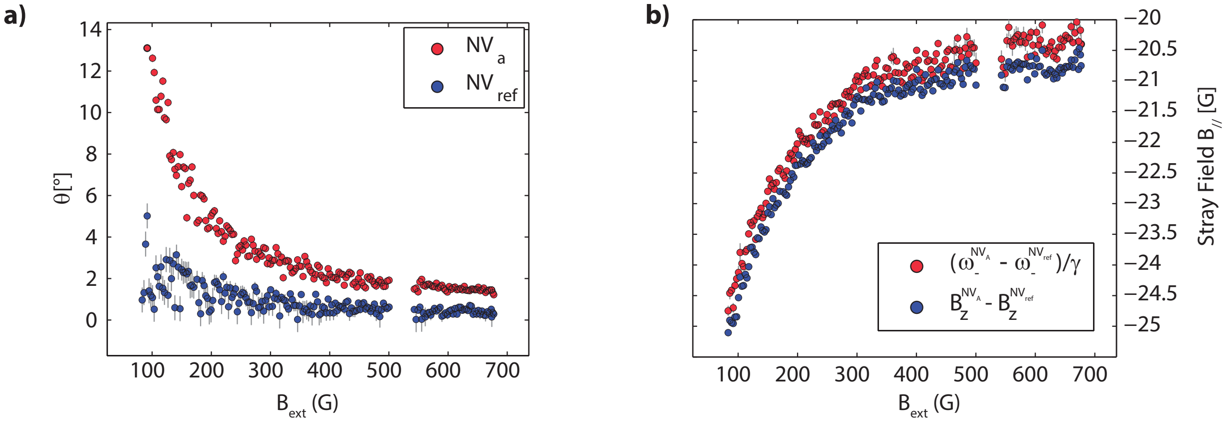

Supplementary Figure 11. **Test of the field alignment.** **a,** Misalignment angle $\theta = \arctan(B_\perp/B_z)$, evaluated for both $\mathrm{NV_{ref}}$ and $\mathrm{NV_a}$. **b,** Stray field $B_z^{\mathrm{NV_a}} - B_z^{\mathrm{NV_{ref}}}$ at the $\mathrm{NV_a}$ (see text), as a result of the presence of the Permalloy disc. The correction for the spatial field gradient from the permanent magnet is not included in the plot.

Supplementary Note 1. Setup and sample

Our experiments were performed on a type IIa diamond grown by chemical vapor deposition (by the company Element 6) measuring 2x2x0.05mm^3. We studied NV centres formed by N^{15} ion implantation at 18keV and a density of 30/μm^2 and subsequent annealing for 2 hours at 800°C, yielding NV centres at an estimated ∼50 nm below the diamond surface.

The magnetic fields used to control the NV centre spin state and to drive spin-wave excitations in the disc were generated by microwave (MW) currents. These currents were delivered to the sample by a Ti/Au 5nm/95nm coplanar waveguide (CPW) with a gap size of 15μm fabricated on the surface of the diamond. In the centre of the CPW gaps, we fabricated permalloy ($Ni_{81}Fe_{19}$) microdiscs by e-beam lithography and subsequent e-beam evaporation. The diamond was glued on a coverslip using optically transparent wax and NV centres were optically accessed through the coverslip-diamond stack with an NA=1.25 oil-immersion objective, resulting in the geometry depicted in Fig. 1a of the main text.

Laser pulses for optical spin initialization and readout were generated by an acousto-optical modulator (AOM) in double-pass configuration. The NV centre spin state was read out by integrating the first 550 ns of photoluminescence during a laser pulse. To avoid melting of the Permalloy disc, we limited the laser power to 250 μW.

The MW bursts used to control the NV spin state and to excite spin-waves in the disc were generated by modulating the output of a MW source (Rohde&Schwarz SMB100a or Agilent N5183A) using pulses generated by an arbitrary waveform generator (Tektronix AWG520) input into a MW switch (Minicircuits ZASWA-2-50DR+ or RFLambda RFSPSTA0208G). To control the NV centre along two orthogonal axes (see Fig. 3a of main text), an additional IQ mixer was incorporated (Marki IQ1545). After amplification (Minicircuits ZVE-3W-83+ 2-8 GHz and ZHL-16W-43+ 1.8-4 GHz), signals from different MW sources were combined using a diplexer (K&L microwave 5IZ10-3050) and fed to the on-diamond CPW through a printed circuit board.

For all the experiments, a static magnetic field was applied that separates the two NV centre spin transitions, which allowed the individual addressing of a target transition by MW pulses at the associated ESR frequency. To assure good optical spin contrast, the static field was carefully aligned with the NV centre crystal axis, using a procedure described in section Supplementary Note 7.

Supplementary Note 2. Numerical simulations

In this section we describe the numerical calculations of the spatial magnetization profile used in this work. Fig. 1f of the main text presented such calculations for the *static* magnetization of the disc. Fig. 2b of the main text shows the numerically calculated ferromagnetic resonance of the disc, and in Fig. 3 of the main text we presented numerical calculations of the time-averaged decrease in longitudinal magnetization of the disc.

A. Static magnetization and conventions

Micromagnetic simulations have been performed with the open source software OOMMF[16] running on the Harvard Odyssey cluster. For all the results presented in this work, the magnetic properties of the Permalloy disc have been simulated imposing a spatial discretization of 5×5×30 nm^3. As in previous works,[20, 21] we chose a saturation magnetization for Permalloy of M_s = 800 kA/m, an exchange coupling of A=10^{-11} Jm^{-1}, a Gilbert gyromagnetic ratio $\gamma = 2.21 \times 10^5$ m/As, a damping constant $\alpha = 0.005$. For the calculation of the static magnetic properties, the value of α has been increased to $\alpha = 0.95$ to favour convergence.[16]

Since the magnetic properties of a microdot are hysteretic [20], all measurements were done by first applying a large field and then sweeping the field down. To obtain Fig. 1e of the main text, we calculated the magnetization profile via the ODE solver of the Landau-Lifshitz equation. Then we calculated the associated disc stray field at the sites of the two NVs. For the calculation of the magnetization we imposed, at the largest computed field of 700 G, a field-aligned spin structure as initial configuration. Subsequently, the initial configuration at a lower field was chosen as the ground state of the adjacent field of bigger magnitude. In order to resemble experimental conditions, the external field orientation in the simulations has been chosen to be the same one of the nitrogen-vacancy center, e.g. along the $[\sin(\theta_{\mathrm{NV}}), 0, \cos(\theta_{\mathrm{NV}})]$ direction, where the z-axis is the one normal to the magnet's surface and $\theta_{\mathrm{NV}} = \arccos(1/\sqrt{3})$. In Supplementary Fig. 1 we show two examples of simulated magnetization patterns (left panels) and the associated magnetic stray field in the NV-plane 50 nm from the disc (right panels).

For the calculation of the stray field created by a certain magnetization pattern (see e.g.

Fig. 1e of the main text) at the site $\mathbf{r}_0$ we used eq. (2) of the main text. It is worth noting that even having perfect knowledge of the stray field $\mathbf{B}(\mathbf{r}_0)$ in the entire plane at fixed distance d from the magnetic film, a unique reconstruction of the static spin structure is not possible, since any additional spin texture $\mathbf{S}(\boldsymbol{\rho})$ for which $\nabla \cdot \mathbf{S}(\boldsymbol{\rho}) = 0$ will not affect the field. Accordingly, the kernel matrix present in eq. (5) of the main text is not invertible.

B. Uniform dynamics

Here we describe how we calculate and define the ferromagnetic resonance (FMR) frequency of the disc as a function of the external magnetic field B_{ext}. We computed the linear, frequency-dependent, spatially-averaged, transverse magnetic susceptibility[15] $\chi''_{\perp}(\omega)$ by applying a spatially uniform magnetic pulse $h(t)$ of the duration $\Delta t = 50 \times 10^{-12}$ s oriented orthogonally to the plane of the disc (resembling the direction of the field generated by our coplanar waveguide). The following time-evolution $m_{\perp}(t)$ of the spatially integrated transverse disc's magnetization (transverse to the applied field and in the disc's plane) was then recorded for a total time of 20 ns, at 5×10^{-12} s time intervals. We computed the field-dependent $\chi''_{\perp}(\omega)$ via a Fast Fourier transform $\mathscr{F}$, using the algorithm:

$$\chi''_{\perp}(\omega) = \mathrm{Im}\left(\frac{\mathscr{F}(m_{\perp}(t) - m_{\perp}(0))}{\mathscr{F}(h(t))}\right), \tag{1}$$

with $h(t)$ expressed in Tesla and $m_{\perp}(t)$ in units of M_s. The numerically calculated quantity $\chi''_{\perp}(\omega)$ differs from Supplementary Eq. (31) in that beyond exchange interactions it includes the effects of dipolar demgnetization fields, which substantially modify the susceptibility at wavelengths comparable with the size of the disc. A colormap combining spectra at several fields is plotted in the left panel of Fig. 2. Green triangles mark the position of the maximum spatially-averaged response, which represents the Kittel's law of the FMR for the disc. Spectral power at two selected fields is shown in the right part of Fig. 2. Well-isolated modes besides the uniform one can be recognized, along the lines of what was shown in previous studies of Permalloy micromagnets.[21]

C. Non-linear spin dynamics

To obtain the calculations shown in Fig. 3c-d of the main text, we used micromagnetics simulations. The non-resonant spin-wave detection scheme (Fig. 3a of the main text)

is sensitive to the time-averaged change $\langle \delta m_{\|,i}(t) \rangle$ of the **longitudinal** magnetization at the local i-site. To extract $\langle \delta m_{\|,i}(t) \rangle$, we numerically average the quantity $\delta m_{\|,i}(t)$ in time, during a continuous excitation of the form $h(t) = h_0 \sin(\omega t)$. The quantity $\delta m_{\|,i}(t)$ represents a non-linear response of the system, because it is not oscillating in time with the same frequency as the excitation field.

In formulas, for the time evolution of the local (site-index i) magnetization $\mathbf{m}_i(t)$, we assumed the following ansatz:

$$\mathbf{m}_i(t) = \mathbf{u}_{\|,i} \left(m_{\|,i}^{\mathrm{eq}} + \delta m_{\|,i}(t) \right) + \sum_{\eta} \mathbf{u}_{\eta,i} m_{\eta,i}(t), \tag{2}$$

Here, $\mathbf{u}_{\|,i}$ is the unit vector locally (index i) parallel to the equilibrium magnetization, and $m_{\|,i}^{\mathrm{eq}}$ is the value of the local equilibrium magnetization. Furthermore, η labels the two axes orthogonal to $\mathbf{u}_{\|,i}$ and $\delta m_{\|,i}(t)$ is the non-linear change of the longitudinal magnetization. Provided with the previous assumption, we extracted from the numerical results the quantity $\delta m_{\|,i}(t)$ by using the algorithm:

$$\delta m_{\|,i}(t) = (\mathbf{m}_i(t) - \mathbf{m}_i^{\mathrm{eq}}) \cdot \mathbf{u}_{\|,i}. \tag{3}$$

The quantity $\delta m_{\|,i}(t)$ is then time-averaged to compute the stray field along the NV axis $\Delta B_{\|}(\omega)$, to which our measurements are sensitive. The quantity $\Delta B_{\|}(\omega)$ is then casted into mG/G^2 by normalizing with the Rabi field $h_0 \sin(\theta_{\mathrm{NV}})/\sqrt{2}$. In the left part of Fig.3 we plot the exemplary in-plane component $\Delta M_{\|}$ of the time-averaged quantity $\langle \delta m_{\|,i}(t) \rangle \mathbf{u}_{\|,i}$ at the frequency ω_{FMR} of the uniform mode. The resulting stray field $\Delta B_{\|}(\omega_{\mathrm{FMR}})$ is shown in the right part of Fig.3. Similar plots were shown in Fig. 3c of the main text.

Supplementary Note 3. Model for the Fano interference in the Rabi oscillations

In this section we describe the fitting procedure for the Fano resonances observed in Fig. 2 of the main text. We use a phenomenological model by considering the interference between the response of a linear system and its driving force. In particular, we use the analogy between the response of a ferromagnet to a driving field and the response of a simple mechanical harmonic oscillator. In both cases energy is stored at resonance; the coherent precession of the two spin components transverse to the equilibrium axis of the ferromagnet is analogous to the periodic transformation of kinetic into potential energy. Furthermore, energy dissipation occurs via spin damping in the ferromagnet and through friction in the oscillator. Finally, both systems are characterized by a phase delay in their response upon driving. Let's consider the equation of motion for a simple mechanical oscillator:

$$m\ddot{x}(t) + \beta\dot{x}(t) + m\Delta^2 x(t) = F(t), \tag{4}$$

where m is the particle's mass, $F(t)$ the driving force, β the friction, Δ the resonance frequency and $x(t)$ the time-dependent coordinate. The oscillator's response in frequency space reads as:

$$x(\omega) = \chi(\omega)F(\omega) = \frac{F(\omega)}{m(\Delta^2 - \omega^2) + i\beta\omega}, \tag{5}$$

where $\chi(\omega)$ is the dynamical susceptibility. Similarly, the transverse dynamical magnetic uniform susceptibility $\chi_\perp(\omega)$ for a ferromagnet with short-range exchange interactions reads:[17]

$$\chi_\perp(\omega) = \frac{g(S)}{(\Delta - \omega) + iW}, \tag{6}$$

where W is the FMR line width, Δ the FMR frequency and $g(S)$ a constant prefactor which depends on the spin quantum number. A Fano interference is created in the mechanical system described by (5) when considering the total response:

$$x_{\text{tot}}(\omega) = (1 + \chi(\omega))\,F(\omega) = \left(1 + r(\omega)e^{i\theta(\omega)}\right)F(\omega), \tag{7}$$

which implies a quadrature summation of the total normalized output's amplitude:

$$\left|\frac{x_{\text{tot}}(\omega)}{F(\omega)}\right| = \sqrt{1 + r^2(\omega) + 2r(\omega)\cos(\theta(\omega))}. \tag{8}$$

Supplementary Eq. (8) is the mechanical equivalent of the expression we have used to model the enhanced normalized Rabi frequency. In particular, in the magnetic case, by calling the

total transverse field at the NV i-site $b^i(\omega)$ and the external driving field $b_D(\omega)$ we obtain:

$$\left|\frac{b^i(\omega)}{b_D(\omega)}\right| = \sqrt{1 + r^2(\omega) + 2r(\omega)\cos(\theta(\omega) + \phi_i)}, \tag{9}$$

where ϕ_i is an additional frequency-independent phase factor motivated by the fact that the stray field created by the ferromagnet varies according to the different NV i-site. The terms $\theta(\omega)$ and $r(\omega)$ can be obtained by taking argument and norm of the complex susceptibility in Supplementary Eq. (6). It is important to note that in order to model our field-dependent normalized Rabi curves (main-text Fig. 2b), one has to impose for the FMR resonance the Kittel-like expression $\Delta = \gamma\sqrt{B_{\text{ext}}(B_{\text{ext}} + A)}$ and for the frequency $\omega = D + \gamma\left(B_{\text{ext}} + B_{\parallel}(B_{\text{ext}})\right)$, where D is the zero field splitting and $B_{\parallel}$ is the projection along the NV axis of the ferromagnet's static stray field (as measured in main-text Fig. 1c). From the fits shown in Fig. 2b of the main text, we extract $W/h = 0.2$ GHz.

Supplementary Note 4. AC Stark effect in off-resonant detection scheme (Fig. 3 of main text)

In this section, we analyze the phase shift imparted on the NV spin state by the AC Stark effect caused by the off-resonant driving field in the measurement scheme of Fig. 3A of the main text. The goal is to estimate if the Stark shift contributes significantly to the measured B_{eff} in Fig. 3 of the main text, taking into account that the AC drive field can be enhanced by the AC field generated by the ferromagnet as was shown in main-text Fig. 2.

The Stark shift, and the related Bloch Siegert shift, [5, 6] is usually treated within an effective two-level model.[4, 5] However, in our experiments, we prepare the $S = 1$ spin of the NV centre in a superposition of $m_s = 0, -1$ states, while the off-resonant driving has a frequency in the vicinity of the $m_s = 0 \leftrightarrow +1$ transition. Therefore, the three-level nature of the system has to be retained.

To discuss the effective field which is picked up by the NV centre as a result of the Stark shift, we employ the time-dependent Schrieffer-Wolff formalism.[7] The method outlined below can be readily generalized to other multilevel quantum systems.

A. Time-Dependent Schrieffer-Wolff formalism

The time-dependent Schrieffer-Wolff formalism is discussed in detail in Ref. 7. Here we briefly recall the main results. We assume an Hamiltonian composed of two parts, a time-independent diagonal part H_0 and a time-dependent non-diagonal part $\mathscr{H}_{nd}(t)$:

$$\mathscr{H}(t) = \mathscr{H}_0 + \mathscr{H}_{nd}(t). \tag{10}$$

In order to derive an effective Hamiltonian $\mathscr{H}_{eff}$ which retains up to second order the effects of the perturbation $\mathscr{H}_{nd}(t)$, we change the quantum basis by applying a unitary transformation $\hat{U}(t) = e^{\hat{S}(t)}$, with $\hat{S}(t) = -\hat{S}(t)^\dagger$.

In the Schrieffer-Wolff formalism, a series expansion of $\hat{S(t)}$ leads to the expression for $\mathscr{H}_{eff}$, which reads:[7]

$$\mathscr{H}_{eff} = \mathscr{H}_0 + \frac{1}{2}\left[\hat{S}(t), \mathscr{H}_{nd}\right] + O(\mathscr{H}_{nd}^3). \tag{11}$$

The matrix $\mathbf{S}(t)$ can be computed from the condition that eliminates the first-order terms $O(\mathscr{H}_{nd})$, namely:[7]

$$\mathscr{H}_{nd}(t) + \left[\hat{S}(t), \mathscr{H}_0\right] + i\dot{\hat{S}}(t) = 0. \tag{12}$$

We will now use this formalism to obtain an expression for the time-independent part of $\mathscr{H}_{eff}$ for an NV centre in an off-resonant driving field.

B. Description of the AC Stark shift for an NV centre in an off-resonant driving field

For an NV centre in a longitudinal static field B_z and a transverse dynamic field B_2, the Hamiltonian reads:

$$\mathscr{H}(t) = \mathscr{H}_0 + \mathscr{H}_{nd}(t) = D(\hat{S}^z)^2 + h\hat{S}^z + h_2 \cos(\omega t)\hat{S}^x, \tag{13}$$

where $S = 1$ spin matrices are used, and we have defined $h = \gamma B_z$ and $h_2 = \gamma B_2$. We assume for $\hat{S}(t)$ the following 3x3 matrix representation, which satisfies the $\hat{S}(t) = -\hat{S}(t)^\dagger$ condition:

$$\hat{S}(t) = \begin{pmatrix} 0 & S_1(t) & 0 \\ -S_1^*(t) & 0 & S_2(t) \\ 0 & -S_2^*(t) & 0 \end{pmatrix}. \tag{14}$$

Provided with Supplementary Eq. (12), the previous expression for $\hat{S}(t)$ defines a set of two linear non-homogeneous differential equations:

$$i\dot{S}_1(t) - \omega_+ S_1(t) + h_2 \cos(\omega t)\frac{\sqrt{2}}{2} = 0 \qquad i\dot{S}_2(t) + \omega_- S_2(t) + h_2 \cos(\omega t)\frac{\sqrt{2}}{2} = 0 \tag{15}$$

where we have defined $\omega_\pm = D \pm h$, which are the spin transition frequencies in the absence of the Stark shift. We compute the solution to Supplementary Eq. (15) imposing $S_{1,2}(t = 0) = 0$, i. e., $\hat{U}(t = 0) = \hat{I}$. We obtain:

$$\begin{aligned} S_1(t) &= -h_2 \frac{\omega_+ e^{-i\omega_+ t} - \omega_+ \cos(\omega t) + i\omega \sin(\omega t)}{\sqrt{2}(\omega_+ - \omega)(\omega_+ + \omega)} \\ S_2(t) &= h_2 \frac{\omega_- e^{-i\omega_- t} - \omega_- \cos(\omega t) - i\omega \sin(\omega t)}{\sqrt{2}(\omega_- - \omega)(\omega_- + \omega)} \end{aligned} \tag{16}$$

By using Supplementary Eq. (11), the effective Hamiltonian can be written as:

$$\mathscr{H}_{eff} \approx \mathscr{H}_0 + \frac{1}{2}\left[\hat{S}(t), \mathscr{H}_{nd}\right] = \mathscr{H}_0 + h_2 \cos(\omega t)\frac{\sqrt{2}}{2}\begin{pmatrix} \frac{S_1(t)+S_1^\dagger(t)}{2} & 0 & \frac{S_1(t)-S_2(t)}{2} \\ 0 & \frac{S_2(t)+S_2^\dagger(t)}{2} - \frac{S_1(t)+S_1^\dagger(t)}{2} & 0 \\ \frac{S_1^\dagger(t)-S_2^\dagger(t)}{2} & 0 & -\frac{S_2(t)+S_2^\dagger(t)}{2} \end{pmatrix}. \tag{17}$$

The resulting time-independent part $\bar{\mathscr{H}}_{eff}$ of the Hamiltonian (17) has the following form:

$$\bar{\mathscr{H}}_{eff} = \mathscr{H}_0 + \begin{pmatrix} A & 0 & \frac{A-B}{2} \\ 0 & B-A & 0 \\ \frac{A-B}{2} & 0 & -B \end{pmatrix}, \qquad A = \frac{h_2^2\omega_+}{4(\omega_+ - \omega)(\omega_+ + \omega)} \qquad B = \frac{-h_2^2\omega_-}{4(\omega_- - \omega)(\omega_- + \omega)}. \tag{18}$$

Note that this effective Hamiltonian contains, in second order, off-diagonal terms that couple directly the $m_s = \pm 1$ subspace. However, in our experiments, the degeneracy of the $m_s = \pm 1$ states is lifted by the static external field h, which represents the dominant energy scale of the subspace. For this reason, as long as $h \gg h_2$, the spin dynamics will mainly be governed by the diagonal elements of $\bar{\mathscr{H}}_{eff}$. In the limit $h \gg h_2$ we are therefore allowed to write:

$$\bar{\mathscr{H}}_{eff} \approx \mathscr{H}_0 + \begin{pmatrix} A & 0 & 0 \\ 0 & B-A & 0 \\ 0 & 0 & -B \end{pmatrix}$$

which describes the shift of the energy levels of the NV spin in the presence of an off-resonant driving field.

C. Analysis of the Stark effect in the off-resonant detection scheme

We now consider the magnetometry sequence shown in Fig. 3a of the main text. The first $\pi/2$ pulse creates a superposition of $m_s = 0$ and $m_s = -1$ states, and all further NV-pulses are also given on the $m_s = 0 \leftrightarrow -1$ transition. The normalized contrast is given by:

$$\begin{aligned} C(\tau, \omega) &= |\langle 0|\Psi\rangle|^2 \\ &= \langle 0|U_y(\pi/2)\exp(-i\mathscr{H}_0\tau/2)U_y(\pi)\exp(-i\bar{\mathscr{H}}_{eff}(\omega)\tau)U_y(\pi)\exp(-i\mathscr{H}_0\tau/2)U_x(\pi/2)|0\rangle|^2. \end{aligned} \tag{19}$$

where $U_{x,y}(\pi/2)$ denotes the operator for a $\pi/2$-pulse around the x, y axis. We obtain the following result:

$$C(\tau,\omega) = \frac{1}{2}\left(1 + \sin\left[\frac{h_2^2\tau}{8}\left(\frac{1}{\omega_+ - \omega} + \frac{2}{\omega_- + \omega} + \frac{2}{\omega_- - \omega} + \frac{1}{\omega_+ + \omega}\right)\right]\right) \tag{20}$$

For illustrative purposes, we now consider two limiting cases of small detuning. If we apply off-resonant driving near the $m_s = 0 \leftrightarrow +1$ transition at $\omega = \omega_+ + \delta$, similar to Fig. 3b-c of the main text, we get

$$C(\tau,\omega) \underset{\omega\to\omega_+ + \delta}{\approx} \frac{1}{2} + \frac{1}{2}\sin\left(\frac{h_2^2}{8\delta}\tau\right). \tag{21}$$

for small δ.

On the other hand, if we would have applied driving near the $m_s = 0 \leftrightarrow -1$ transition at $\omega = \omega_- + \delta$, we get

$$C(\tau,\omega) \underset{\omega=\omega_- + \delta}{\approx} \frac{1}{2} + \frac{1}{2}\sin\left(\frac{h_2^2}{4\delta}\tau\right). \tag{22}$$

which is the same as obtained from a two-level treatment of the NV-centre[5]. In this case, the Stark shift is twice that of Supplementary Eq. (21) because the driving is applied near the transition frequency of the two states forming the superposition.

D. Comparing the full Stark-effect model with experiments

As a check of our model, we now quantitatively compare Supplementary Eq. (20) with experiments, applying the measurement scheme in Fig.3a of the main text to NV_{ref}. In the first experiment, we apply all NV control pulses at the $0 \leftrightarrow +1$ transition. In the second experiment we apply all NV pulses on the $0 \leftrightarrow -1$ transition. In both experiments, we apply off-resonant driving close to the $0 \leftrightarrow +1$ transition at a frequency $\omega = \omega_+ + \delta$, with $\delta > 0$.

Supplementary Fig. 4 shows the accumulated Stark phase ϕ in terms of an effective field $B_{eff} = \phi/(\gamma T)$, where T is the duration of the off-resonant driving. We normalized B_{eff} by the square of the amplitude of the driving field B_2^2 to correct for a frequency-dependence in the setup transmission. We independently measured $B_2(\omega)$ by measuring the Rabi frequency $\omega_\mathrm{R}(\omega)$ of NV_{ref} as a function of ESR frequency ω which we vary using B_{ext}. Note that for an NV centre it can be shown[19] that $\omega_\mathrm{R} = B_2/\sqrt{2}$. We find a good agreement with Supplementary Eq. (20) (solid lines) for both experiments. We indeed observe a difference in the Stark effect according to which set of spin states we apply the NV-control pulses.

The dotted line shows the approximation given in Supplementary Eq. (21), which should be compared to the red solid line.

E. Estimate of the 'enhanced' Stark effect in the measurements of Fig. 3

In this section, we estimate the magnitude of the Stark effect at the frequency of the FMR in Fig. 3 of the main text. The Stark effect quickly diminishes with increasing detuning, which is visible in Fig. 3c of the main text in the frequency range just above the $0 \leftrightarrow +1$ transition over the entire magnetic field range. However, as we observed that the drive field may be enhanced by the spin-wave field (Fig. 2 of the main text), an 'enhanced' Stark effect may contribute to the observed resonances in Fig. 3 of the main text, provided the enhancement is strong enough.

For the plot in the right part of Fig. 4, we use the experimentally observed $\approx$doubling of the Rabi frequency and plot a Stark field four times as big as the one measured in the left part of the figure. We conclude that the Stark effect is negligible for the resonance observed on NV_B (top panel of Fig. 3b of main text) which is on the order of 100 mG/G^2. On the other hand, for NV_A (Fig. 3b of main text, bottom panel), the estimated Stark effect at the FMR is on the same scale as the signal in the low-field range $B_{ext} \sim< 300$ G. However, the negative sign of B_{eff} in this range cannot result from the Stark effect (the sign will be described in section Supplementary Note 4 F). For $B_{ext} > 300$ G, the estimated Stark effect is much smaller than the observed signal.

F. Sign of the effective field associated with the Stark shift

Finally, we discuss how the Stark shift provides a convenient way to assign a sign to the effective field measured with the scheme of Fig. 3a of the main text.

To see if the Stark shift corresponds to a positive or negative effective field B_{eff}, we need to consider the Hamiltonian in Supplementary Eq. (19). As an example, we assume as in our experiments that $\omega = \omega_+ + \delta$, with $\delta > 0$, which leads to $A < 0$ while $B \approx 0$. Suppose the NV is prepared in a arbitrary superposition $\Psi(t=0) = a|0\rangle + b|-1\rangle + c|+1\rangle$. The time evolution in a field h and with $A < 0$ will read as:

$$\Psi(t) = e^{iAt}\left(a|0\rangle + be^{-i(D-h-|A|)t}|-1\rangle + ce^{-i(D+h-2|A|)t}|+1\rangle\right). \quad (23)$$

It is evident from Supplementary Eq. (23) that the Stark shift corresponds to a *positive* effective field if we create a superposition of the 0,-1 states (with $a, b \neq 0, c = 0$) because the field h has changed to $h + |A|$. This positive Stark field corresponds to the experimental case. On the other hand, the effective field is *negative* for a superposition $a, c \neq 0, b = 0$, because the field h has changed to $h - 2|A|$. Therefore, the observation of the Stark shift, which as mentioned before is visible in Fig. 3c of the main text in the frequency range just above the $0 \leftrightarrow +1$ transition, allows us determine the sign of B_{eff} in both the measurements in Fig. 3b-c of the main text (these measurements used the exact same pulse sequence).

Supplementary Note 5. Normalization procedure for the off-resonant detection scheme (Fig. 3 of main text)

In this section we describe the normalization procedure used to obtain Figs. 3b,d of the main text.

We use the measurement scheme shown in Fig. 3a of the main text, in which we apply the final $\pi/2$-pulse along the x- or y-axis and subsequently read out the spin-dependent PL (P_x and P_y resp.). During the same measurement, we also apply two normalization sequences which are the same as in Fig. 3a of the main text except that we turn the MW off and apply the final $\pi/2$-pulse along the x and $-x$ axis. Supplementary Fig. 5a shows the raw data of these four measurements. The normalization sequences yield the minimum and maximum PL (P_{min} and P_{max} resp.) which we use to obtain the x and y spin expectation values (Supplementary Fig. 5b) according to $\langle i \rangle = 2\frac{P_i - P_{min}}{P_{ax} - P_{min}} - 1$, where $i = x, y$.

From the expectation values, we calculate the phase ϕ of the superposition using $\phi = \arctan(\frac{\langle y \rangle}{\langle x \rangle})$ (Supplementary Fig. 5c). We express the final signal in terms of an effective field $B_{eff} = \phi/(\gamma T)$, and divide by the square of the driving field $|b_d|^2$ to correct for the frequency-dependent delivery of microwaves through our setup (Supplementary Fig. 5d). We independently characterized $|b_d|^2$ by measuring the Rabi frequency of NV_{ref} at constant MW-source power as a function of the ESR frequency, which we tune using B_{ext} (Supplementary Fig. 6). The inset of Supplementary Fig. 5c illustrates the effect of the frequency-dependent power on the measurement of ϕ: dips/peaks in ϕ clearly correspond to dips/peaks in the Rabi frequency, motivating the normalization by $|b_d|^2$.

To validate the procedure of normalizing B_{eff} by the square of the drive field $|b_d|^2$ (Supplementary Fig. 5d), we studied the dependence of B_{eff} on the power of the MW source R, since $|b_d|^2 \propto R$. Supplementary Fig. 7 shows the linear scaling of the signal with MW power.

Supplementary Note 6. Stray-field characterization of magnetization and spin noise

In this section we first describe the fitting procedure used to extract the relaxation rates from the measurements in Fig. 4a of the main text. Then we describe the model linking the relaxation rates to the spin-noise created by the disc. The following two sections are complementary to what discussed in the Methods section of the main text.

A. Noise probed by an NV centre

To describe the NV-spin relaxation we use a rate-equation model[13]:

$$\frac{\mathrm{d}\mathbf{P}(t)}{\mathrm{d}t} = \bar{\mathbf{W}}\mathbf{P}(t) = \begin{pmatrix} -(W_{1,0}+W_{-1,0}) & W_{-1,0} & W_{1,0} \\ W_{-1,0} & -(W_{-1,0}+W_{1,-1}) & W_{1,-1} \\ W_{1,0} & W_{1,-1} & -(W_{1,-1}+W_{1,0}) \end{pmatrix} \mathbf{P}(t) \quad (24)$$

where $\mathbf{P}(t)$ describes the populations of the three NV-spin states as a function of time. In our fitting procedure, we imposed $W_{1,-1} = 0$. This is validated by noting that magnetic-field noise does not directly couple the $m_s = -1, 1$ levels. In addition, we observed that the relaxation of NV_A is dominated by magnetic-field noise (it has a much faster relaxation than far-away NV_{ref} which we confirmed in a separate measurement to be ~1/ms). The relaxation dynamics is therefore described by only two parameters: $W_{1,0}$ and $W_{-1,0}$.
In second order perturbation theory, the relaxation parameters are given by:[14]

$$W_{\alpha,\beta} \underset{\alpha\neq\beta}{=} \frac{1}{\hbar^2}\int_{-\infty}^{\infty} \mathrm{d}\tau e^{i\omega_{\alpha,\beta}\tau}\overline{\langle\alpha|\mathscr{H}_1(\tau)|\beta\rangle\langle\beta|\mathscr{H}_1(0)|\alpha\rangle}, \quad (25)$$

where the $|\alpha\rangle$, $|\beta\rangle$ are the spin eigenstates of the NV centre and $\hbar\omega_{\alpha,\beta}$ is the energy difference between the levels ($\omega_{\alpha,\beta} = (\omega_\alpha - \omega_\beta)$). The time-dependent magnetic perturbation at the NV centre due to magnetic-field fluctuations can be written as:

$$\mathscr{H}_1(\tau) = \hbar\gamma \sum_\eta \bar{I}_\eta \delta B_\eta(\tau), \quad (26)$$

where $\bar{I}_\eta$ is the η-component of the spin operator of the $I = 1$ spin of the NV and $\gamma = 2\pi\cdot 28$ GHz/T. It is easy to show that, by defining:

$$g(\omega_{\alpha,\beta}) = \frac{\gamma^2}{2}\int_{-\infty}^{\infty} \mathrm{d}\tau e^{i\omega_{\alpha,\beta}\tau} \sum_{\eta\neq z} \overline{\delta B_\eta(\tau)\delta B_\eta(0)} = \frac{\gamma^2}{2}|B_\perp(\omega_{\alpha,\beta})|^2, \quad (27)$$

we have:

$$W_{1,0} = g(\omega_{+1} - \omega_0) \qquad W_{-1,0} = g(\omega_{-1} - \omega_0). \tag{28}$$

Besides probing the spin-noise spectrum at different frequencies, the two relaxation channels are formally identical.

Combining eq. (8) of the main text and Supplementary Eq. (27), we reach the following general expression for the characteristic relaxation rate of an NV centre in the vicinity of a thin magnetic film:

$$g(\omega_{\alpha,\beta}) = \frac{\gamma^2}{2}\frac{\Gamma^2}{(2\pi)^2}\sum_{\substack{m\neq z\\ n}}\int_{\mathbf{k}} \mathcal{N}^{m,n}(\mathbf{k},d)\cdot \mathbf{S}^n(\omega_{\alpha,\beta},\mathbf{k})\mathrm{d}\mathbf{k}. \tag{29}$$

Finally, note that the T- (temperature) and B_{ext}-dependent spin fluctuations $S^{\eta,\eta}(\omega_{\alpha,\beta},\mathbf{k})$ can be related to the more well-known spin susceptibility $\chi''_{\eta',\eta'}(\omega_{\alpha,\beta},\mathbf{k})$ with the fluctuation-dissipation theorem:[15]

$$S^{\eta,\eta}(\omega_{\alpha,\beta},\mathbf{k}) = \frac{2\hbar}{1-e^{-\beta\hbar\omega_{\alpha,\beta}}}\chi''_{\eta',\eta'}(\omega_{\alpha,\beta},\mathbf{k}) \underset{\hbar\omega_{\alpha,\beta}\ll 1/\beta}{\approx} \cdot\frac{2k_{\mathrm{B}}T}{\omega_{\alpha,\beta}}\chi''_{\eta,\eta}(\omega_{\alpha,\beta},\mathbf{k}) \tag{30}$$

B. Noise due to a two-dimensional ferromagnetic thin film

In this section we evaluate Supplementary Eq. (29) for the case of an infinitely extended 2d ferromagnetic layer of Permalloy. As discussed in the Methods section of the main text, the NV center is mostly sensitive to spin fluctuations with wavenumber $k \sim 1/d$. Because $d \gg a$, where a is the lattice parameter, we can neglect the microscopic details of the lattice. Accordingly, we will compute the magnetic noise from a system composed by an infinitely thin ferromagnetic layer having a magnetic dipole density Γ. This procedure leads to the calculated noise spectrum and associated NV-relaxation rates shown in Fig. 4c of the main text (and to the corresponding solid lines in Fig. 4b of the main text.)

We will give an estimate of the characteristic relaxation time including at first only in-plane transverse spin fluctuations, assuming that at the wavelengths $k \sim 1/d$ the exchange interactions are dominant. Assume the plane is magnetized along the z-axis. The susceptibility for a 2d ferromagnet along one of the axes transverse to z is given by:[17]

$$\chi''_{y,y}(\omega_{\alpha,\beta},k) = \Gamma^{-1}S\frac{W}{W^2 + (Dk^2 + \Delta - \hbar\omega_{\alpha,\beta})^2}, \tag{31}$$

where D is the spin stiffness [17], W the width of the FMR excitation, Δ its energy and S is the value of the local spin. In the previous expression the k in the denominator is expressed in m^{-1}. The value of D can be taken from previous works [18], namely $D = 370$ meVÅ^2. Because of the demagnetization energy cost for out-of-plane spin fluctuations, we can in addition safely assume that $\chi''_{y,y} \gg \chi''_{x,x}$ and include in the calculations only the contribution of $\chi''_{y,y}$.

In the case of Supplementary Eq. (31) the susceptibility has no ϕ_k-dependence. We can therefore integrate out the ϕ_k variable in the integral (29). We define the following θ_{NV}-dependent prefactor:

$$
\begin{aligned}
F(\theta_{\mathrm{NV}}) &= \Big(\frac{g_L \mu_0 \mu_{\mathrm{B}}}{2}\Big)^2 \int_0^{2\pi} \Big(\Big| i \sin(\phi_k)\cos(\theta_{\mathrm{NV}}) + \frac{\sin(2\phi_k)}{2}\sin(\theta_{\mathrm{NV}}) \Big|^2 + \big|\sin^2(\phi_k)\big|^2 \Big) \mathrm{d}\phi_k \\
&= \frac{(g_L \mu_0 \mu_{\mathrm{B}})^2}{32} \pi \Big(11 + 3\cos(2\theta_{\mathrm{NV}}) \Big). \qquad (32)
\end{aligned}
$$

We can now solve the integral (29), which gives:

$$
\begin{aligned}
g(\omega_{\alpha,\beta}) &= \frac{\gamma^2}{2} \frac{\Gamma}{(2\pi)^2} \left(\frac{2k_{\mathrm{B}}T}{\omega_{\alpha,\beta}} \right) S F(\theta_{\mathrm{NV}}) \int_0^{\infty} k^3 e^{-2dk} \frac{W}{W^2 + (Dk^2 + \Delta - \hbar\omega_{\alpha,\beta})^2} \mathrm{d}k \\
&= \frac{\gamma^2}{2} \frac{\Gamma}{(2\pi)^2} \left(\frac{2k_{\mathrm{B}}T}{\omega_{\alpha,\beta}} \right) S F(\theta_{\mathrm{NV}}) \Big[- \frac{W + i(\Delta - \hbar\omega)}{4D^2\sqrt{\pi}} G^{3,1}_{0,0}\left({-1 \atop -1,0,1/2} \,\middle|\, d^2 \frac{-iW + \Delta - \hbar\omega}{D} \right) \\
&\quad - \frac{W - i(\Delta - \hbar\omega)}{4D^2\sqrt{\pi}} G^{3,1}_{0,0}\left({-1 \atop -1,0,1/2} \,\middle|\, d^2 \frac{iW + \Delta - \hbar\omega}{D} \right) \Big], \qquad (33)
\end{aligned}
$$

where the function $G^{m,n}_{p,q}\left({a_1,\ldots,a_p \atop b_1,\ldots,b_q} \,\middle|\, z \right)$ is the Meijer G-function and d is the distance of the NV centre from the ferromagnetic film.

The previous expression formally holds true in the case of an infinitely large and thin magnetic film and it represents the contribution to the magnetic relaxation rate due solely to transverse spin fluctuations of the ferromagnet. On the other hand, longitudinal spin fluctuations take into account thermal intra-band spin-wave transitions;[17] due to the much smaller scale of the Zeeman with respect to the thermal energy, their contribution is essentially field-independent. We include them by summing up the constant $g_{\parallel}$ to the expression (33).

For a comparison with the experimental data we have used $W/h = 0.2$ GHz. This value was extracted from the fits of the Fano interferences in Fig. 2 of the main text with the damped oscillator response in Supplementary Eq. (6). We also vary $\Delta(H)$ as the Kittel law measured in our experiments for a 6 μm large disc and put $S = 1/2$ since fcc Permalloy has

on average one Bohr magneton per site ($M_s \cdot a^3/4 \approx \mu_{\mathrm{B}}$) and a Landé factor $g_L \approx 2$ [22]. Finally, we used $M_s = 8 \cdot 10^5$ A/m as the saturation magnetization.[12] A plot of $g(\omega_{\alpha,\beta})$ with these parameters and $d = 35$ nm is in Supplementary Fig. 8b.

Supplementary Eq. (33) represents the noise produced by a two-dimensional ferromagnetic plane at a distance d. To illustrate the dependence on d, we shows fits to the noise measurements in Fig. 9 (same measurements as in Fig. 4b of the main text), in which we fixed d while fitting the parameters t and the field-independent offset $g_{\|}$. When also releasing d, we obtain the fit for the full field-dependence of the spectrum $g(\omega_{\alpha,\beta})$ shown in Fig. 4b of the main text with $d = 35(5)$ nm, in reasonable agreement with the ~ 50 nm estimated from the implantation energy.

We now discuss the values of the fit parameter t, which underestimates the 30 nm expected for our evaporated Permalloy disc as can be seen from Supplementary Fig. 9. We note that several effects may play a role. Importantly, in our approximation the magnetic film is assumed infinitely thin, thus neglecting the smaller noise produced by spins that are located at a larger distance than the distance d from the NV centre. Furthermore, corrections due to the discretization of the spin wave spectrum associated with the finite thickness of the disc may also change the expected spin noise. Finally, a smaller saturation magnetization, fabrication-related imperfections, and/or oxidation of the disc may also lead to smaller spin noise.

In summary, our model for the spin noise captures the main physics of the problem, introducing a formulation of NV magnetometry in momentum space, which will be of use in future investigations of condensed-matter systems.

Supplementary Note 7. Photoluminescence-based field alignment procedure

In our experiments, we use the spin of the NV centre as an optically interrogated magnetometer. To assure good optical spin contrast, it is essential to align the applied magnetic field B_{ext} with the N-V crystal axis [1]. In our experiments, all field sweeps are conducted by translating a permanent magnet along computed space trajectories that keep B_{ext} aligned with the NV axis. In this section, we describe the procedure we used to find these trajectories, based on combining a model of the field generated by our magnet with a model of the NV-centre photoluminescence described by Tetienne et al[1]. In section Supplementary Note 7 C we describe experimental tests of the quality of the alignment.

A. Calculation of the photoluminescence

To apply B_{ext}, we used cylindrical NdFeB magnets of variable dimensions. We calculate their space-dependent magnetic field profile with the open source Radia package[2]. Furthermore, we calculate the PL of an NV centre in a field using the rate-equation model and transition rates from Ref. 1. Combining these models yields characteristic plots for the space-dependent PL (Fig. 10 left panel). The white dashed line marks the trajectory corresponding to perfect alignment. It is clear that the PL is very sensitive to misalignment whenever the field is ≈ 500 G or ≈ 1000 G. These field values correspond to the level anti-crossing of the ground and excited NV states, respectively.[1]. A direct comparison with experimental data around ≈ 500 G (Fig. 10, right panel) illustrates the quality of the model.

A transformation between the laboratory frame and the model frame is essential for calculating the magnet position corresponding to a given target magnetic field. The procedure to obtain this transformation is described next.

B. Determining the magnet trajectory along which the field is well aligned

To calculate the magnet trajectory along which the field is well aligned with the NV axis, we use the strong PL dependence on the magnet position in the $B_{ext} \geq 514$ G field range, which can be easily identified by the nodal point in the PL (Fig. 10 right panel). In particular, we first translated the magnet along orthogonal directions lying within N different constant-Z

planes, recording the coordinates of a set of $\mathbf{y}_i$ points, $\{i = 1, ..., N\}$, where the PL was found to peak. The $\mathbf{y}_i$ represent a set of magnet positions in the laboratory frame for which the field is aligned. For each of these $\mathbf{y}_i$ points we measured $B_{ext,i}$ by ESR. Provided with $B_{ext,i}$, we numerically compute the corresponding $\mathbf{x}_i$ positions of the magnet in the model frame. The transformation relating the $\mathbf{y}_i$ to the $\mathbf{x}_i$ is:

$$\mathbf{y}_i = \mathbf{R}\mathbf{x}_i + \mathbf{t}, \tag{34}$$

where $\mathbf{R}$ is assumed to be a pure rotation matrix and $\mathbf{t}$ a translation vector. We obtain the matrix $\mathbf{R}$ via Wahba's method[3], which provides an expression for $\mathbf{R}$ based on the minimization of the following cost function:

$$\mathscr{L}(\mathbf{R}) = \frac{1}{2(N-1)} \sum_{i=1}^{N-1} ||(\mathbf{y}_i - \mathbf{y}_1) - \mathbf{R}\left(\mathbf{x}_i - \mathbf{x}_1\right)||^2 . \tag{35}$$

The algorithms for minimizing $\mathscr{L}(\mathbf{R})$ and computing $\mathbf{R}$ are described in Ref. 3. We then obtain the vector $\mathbf{t}$ by:

$$\mathbf{t} = \frac{1}{N} \sum_{i=1}^{N} \left(\mathbf{y}_i - \mathbf{R}\mathbf{x}_i\right). \tag{36}$$

Provided with the matrix $\mathbf{R}$ and the vector $\mathbf{t}$, the laboratory frame coordinates of the magnet can be calculated for any value of $B_{ext,i}$ via (34). The next section describes experimental tests of the quality of the field alignment by measuring the field dependence of the ESR resonances of $\mathrm{NV}_{\mathrm{ref}}$.

C. Test of the field alignment along the numerically calculated magnet trajectory

In order to evaluate the quality of the field alignment along the numerically calculated maganet trajectory, we use single-NV vector magnetometry[11]. We measure the ESR frequencies $\omega_\pm$ of the $m_s = 0 \leftrightarrow \pm 1$ transitions to determine the magnitude of the external field along (B_z) and transverse to ($B_\perp$) the NV axis.

In particular, we consider the NV Hamiltonian:

$$\mathscr{H} = D(\hat{S}_z)^2 + \gamma B_z \hat{S}_z + \gamma B_\perp \hat{S}_x, \tag{37}$$

where D is the zero-field splitting, and $\gamma = 2\pi \cdot 2.8025$ MHz/G is the NV gyromagnetic ratio.

The eigenvalues of Supplementary Eq. (37) are given by the solution of the characteristic equation:

$$||\mathscr{H} - \lambda_i \mathbf{I}|| = 0, \tag{38}$$

$$\lambda_1 = \lambda_0 \qquad \lambda_2 = \lambda_0 + \omega_+ \qquad \lambda_3 = \lambda_0 + \omega_-. \tag{39}$$

We substitute Supplementary Eq. (39) into Supplementary Eq. (38) and solve for $\lambda_0, B_z, B_\perp$, using the measured values for $\omega_\pm$. We obtain:

$$B_z = \frac{\sqrt{-(D+\omega_+ - 2\omega_-)(D+\omega_- - 2\omega_+)(D+\omega_- + \omega_+)}}{3\gamma\sqrt{3D}}, \tag{40}$$

$$B_\perp = \frac{\sqrt{-(2D-\omega_+ - \omega_-)(2D+2\omega_- - \omega_+)(2D-\omega_- + 2\omega_+)}}{3\gamma\sqrt{3D}}. \tag{41}$$

The previous expressions rely on the value of D, which we extracted from the field-independent average $D = (\omega_+ + \omega_-)/2$ to be $D = 2\pi \cdot 2.8707(1)$ GHz. In Fig. 11a we plot the extracted misalignment angle, defined as $\arctan(B_\perp/B_z)$, for both $\mathrm{NV_{ref}}$ and $\mathrm{NV_a}$. A $\theta \neq 0$ value for $\mathrm{NV_{ref}}$ is solely due to a misaligned field of the permanent magnet, which is limited to the small value of $\theta \leq 3°$ (Fig. 11a). For $\mathrm{NV_a}$, the stray field from the Py disc causes the low-field increase of θ.

D. Determining the disc field from the ESR traces

The field created by the permanent magnet varies slightly between the different NVs studied in this work. In addition, the associated field gradient varies also with the magnitude of the external static field applied along the NV centre axis. By measuring the externally applied field at reference NV sites distant from the Permalloy disc, we measure a field gradient of ~ 0.2 G/μm at the maximum static field used in experiments of $B_z^{\mathrm{NV_{ref}}} = 700$ G (applied using the model magnet D48-N52 produced by K&J magnetics). We confirm numerically the strength and field-dependence of the measured field gradient, and the fact that the latter is uniform in space within the optical field of view. In this way, we can compute the field-dependence $|\Delta B_z^{\mathrm{NV_{a,b}}}|$ of the difference between the external static field at the NV sites a, b with respect to the reference NV.

Finally, using the methods described in the previous paragraph, the values of B_z at both $\mathrm{NV_{ref}}$ and $\mathrm{NV_{a,b}}$ as extracted from the ESR resonances allow us to evaluate the residual stray

field $B_{//} = B_z^{\mathrm{NV_{a,b}}} - B_z^{\mathrm{NV_{ref}}} + |\Delta B_z^{\mathrm{NV_{a,b}}}|$ created by the Permalloy disc only. In Supplementary Fig. 11b we compare $B_z^{\mathrm{NV_{a,b}}} - B_z^{\mathrm{NV_{ref}}}$ with the simpler stray field estimate $(\omega_-^{\mathrm{NV_{ref}}} - \omega_-^{\mathrm{NV_a}})/\gamma$. In the main text, we show the field dependence of $B_{//}$, which includes the correction for the field-gradient.

Supplementary References.

[1] J.-P. Tetienne, L. Rondin, P. Spinicelli, M. Chipaux, T. Debuisschert, J.-F. Roch and V. Jacques, *Magnetic-field-dependent photodynamics of single NV defects in diamond: an application to qualitative all-optical magnetic imaging*, New Journ. of Phys. **14**, 103033 (2012).

[2] O. Chubar, P. Elleaume and J. Chavanne, *A 3D Magnetostatics Computer Code for Insertion Devices*, Jour. of Synch. Rad. **5**, 481 (1998).

[3] F. L. Markley and D. Mortari, *How To Estimate Attitude from Vector Observations*, Proceedings: Advances in the Astronautical Sciences **103**, 1979 (1999).

[4] S. Rohr, E. Dupont-Ferrier, B. Pigeau, P. Verlot, V. Jacques, and O. Arcizet, *Synchronizing the Dynamics of a Single Nitrogen Vacancy Spin Qubit on a Parametrically Coupled Radio-Frequency Field through Microwave Dressing*, Phys. Rev. Lett. **112**, 010502 (2014).

[5] P. Cappellaro, L. Jiang, J. S. Hodges, and M. D. Lukin, *Coherence and Control of Quantum Registers Based on Electronic Spin in a Nuclear Spin Bath*, Phys. Rev. Lett. **102**, 210502 (2009).

[6] P. London, P. Balasubramanian, B. Naydenov, L. P. McGuinness, and F. Jelezko, *Strong driving of a single spin using arbitrarily polarized fields*, Phys. Rev. A **90**, 012302 (2014).

[7] H. Wang and G. Burkard, *Mechanically induced spin resonance in a carbon nanotube*, Phys. Rev. B **90**, 035415 (2014).

[8] J. R. Schrieffer and P. A. Wolff, *Relation between the Anderson and Kondo Hamiltonians*, Phys. Rev. **149**, 491 (1966).

[9] S. Poletto *et al.*, *Entanglement of Two Superconducting Qubits in a Waveguide Cavity via Monochromatic Two-Photon Excitation*, Phys. Rev. Lett. **109**, 240505 (2012).

[10] M. Noske, A. Gangwar, H. Stoll, M. Kammerer, M. Sproll, G. Dieterle, M. Weigand, M. Fähnle, G. Woltersdorf, Ch. H. Back, and G. Schütz, *Unidirectional sub-100 ps magnetic vortex core reversal*, Accepted for publication in Phys. Rev. B (2014).

[11] G. Balasubramanian, I. Y. Chan, R. Kolesov, M. Al-Hmoud, J. Tisler, C. Shin, C. Kim, A. Wojcik, P. R. Hemmer, A. Krueger, T. Hanke, A. Leitenstorfer, R. Bratschitsch, F. Jelezko and Jörg Wrachtrup, *Nanoscale imaging magnetometry with diamond spins under ambient conditions*, Nature **455**, 648 (2008).

[12] C. Bayer, J. Jorzick, B. Hillebrands, S. O. Demokritov, R. Kouba, R. Bozinoski, A. N. Slavin, K. Y. Guslienko, D. V. Berkov, N. L. Gorn, and M. P. Kostylev, *Spin-wave excitations in finite rectangular elements of Ni80Fe20*, Phys. Rev. B **72**, 064427 (2005).

[13] A. Jarmola, V. M. Acosta, K. Jensen, S. Chemerisov, and D. Budker, Phys. Rev. Lett. **108**, 197601 (2012).

[14] A. Suter, M. Mali, J. Roos and D. Brinkmann, *Mixed magnetic and quadrupolar relaxation in the presence of a dominant static Zeeman Hamiltonian*, J. Phys. Condens. Matter **10**, 5977 (1998).

[15] F. Schwabl. *Advanced Quantum Mechanics*, Springer, 2005.

[16] M.J. Donahue and D.G. Porter, Nat. Inst. of Stand. and Tech., Gaithersburg, MD (1999).

[17] L. Trifunovic, F. L. Pedrocchi, and D. Loss, *Long-Distance Entanglement of Spin Qubits via Ferromagnet*, Phys. Rev. X **3**, 041023 (2013).

[18] P. Yu, X. F. Jin *et al.*, Phys. Rev. B **77**, 054431 (2008).

[19] See Supp. Mat. of R. Hanson, V. V. Dobrovitski, A. E. Feiguin, O. Gywat and D. D. Awschalom, *Coherent Dynamics of a Single Spin Interacting with an Adjustable Spin Bath*, Science **18**, 352 (2008).

[20] J. P. Davis, D. Vick, J. A. J. Burgess, D. C. Fortin, P. Li, V. Sauer, W. K. Hiebert and M. R. Freeman, *Observation of magnetic supercooling of the transition to the vortex state*, New. Jour. of Phys. **12**, 093033 (2010).

[21] M. Bailleul, R. Höllinger, and C. Fermon, *Microwave spectrum of square Permalloy dots: Quasisaturated state*, Phys. Rev. B **73**, 104424 (2006).

[22] C. Luo, Z. Feng,Y. Fu, W. Zhang, P. K. J. Wong, Z. X. Kou, Y. Zhai, H. F. Ding, M. Farle, J. Du, and H. R. Zhai, *Enhancement of magnetization damping coefficient of permalloy thin films with dilute Nd dopants*, Phys. Rev. B **89**, 184412 (2014)
